# Joint Aging Patterns in Brain Function and Structure Revealed Using 27,793 Samples

**DOI:** 10.34133/research.0887

**Published:** 2025-09-25

**Authors:** Yuhui Du, Ruotong Li, Ying Xing, Vince D. Calhoun

**Affiliations:** ^1^ School of Computer and Information Technology, Shanxi University, Taiyuan, China.; ^2^ Tri-Institutional Center for Translational Research in Neuroimaging and Data Science (TReNDS), Georgia State University, Georgia Institute of Technology, Emory University, Atlanta, GA, USA.

## Abstract

Aging has important impacts on both the function and structure of the brain, yet the interplay between these changes remains unclear. Here, we present a unified framework including both single-modal and multimodal age predictions using a large UK Biobank dataset (27,793 healthy subjects, 49 to 76 years) to identify and validate brain functional network connectivity (FNC) and gray matter volume (GMV) changes associated with aging, then propose a novel analysis method to reveal various joint aging patterns, and finally investigate the association between joint function–structure changes and cognitive declines. Multimodality outperforms single modality in the age prediction, underscoring the significance of multimodal aging-related changes. Aging primarily induces synergistic changes, with both FNC and GMV decreased in the cerebellum, frontal pole, paracingulate gyrus, and precuneus cortex, indicating consistent degeneration in motor control, sensory processing, and emotional regulation, and contradictory changes with increased FNC magnitude but decreased GMV in the occipital pole, lateral occipital cortex, and frontal pole, acting as a compensatory mechanism as one ages to preserve visual acuity, cognitive ability, and behavioral modulation. Particularly, joint changes, with both FNC and GMV decreased in the crus I cerebellum and the paracingulate gyrus, show a strong Pearson correlation with the reaction time. In summary, our study unveils diverse joint function–structure changes, providing strong evidence for understanding distinct cognitive deteriorations during aging.

## Introduction

Brain aging of healthy populations, which notably affects various cognitive functions [1–4], stands out as a severe aspect of aging. Cognitive decline is related to changes in brain structure of specific brain areas [5–9] or results from altered functional interaction between brain areas [10,11]. Noninvasive neuroimaging techniques, such as structural magnetic resonance imaging (sMRI) and functional magnetic resonance imaging (fMRI), have been applied to the exploration of brain changes during aging [5,6,12,13]. Previous studies utilizing sMRI data have mainly found age-related changes in gray matter volume (GMV) [14–16]. FMRI studies have revealed that resting-state functional connectivity shows both increases and decreases as people age [12,17,18], spanning large-scale brain networks [18–21]. Indeed, there is a common belief that changes in brain function and structure during aging occur simultaneously, as these changes are often interconnected under various situations [22] such as developmental progression, disease conditions, brain plasticity activity, and brain stimulus treatment [23,24]. In this work, we use multimodal analyses to investigate how brain function and structure are linked with aging, and how these changes affect cognitive function.

There have been multiple efforts to construct age prediction models by combining brain structural and functional imaging data [25–27], thereby exploring aging-related multimodal changes [1,13,28,29]. However, previous multimodal age prediction studies often directly concatenate features from different modalities as input to a unified prediction model [28,30,31], which may inadvertently prioritize brain structural measures over functional ones, given that brain structural features typically exhibit more pronounced age-related changes than functional measures [29,32–34]. The processing approach allows the structurally dominant features to overshadow more subtle functional contributions, thus hindering further investigation into how structure and function jointly contribute to brain aging. While employing the leave-one-modality-out method can help compare the performance of models across different modalities in age prediction [31], identifying age-related joint changes across multiple modalities poses a challenge. Some work used a 2-level model for age prediction in which single-modality models were first trained to predict age, and their output ages were then used for training the second-level model to obtain the fused age [34]. However, the 2-level model complicates the identification of multimodal changes that are reliably and directly associated with aging. Apart from utilizing age prediction, independent component analysis (ICA) [13] has also been applied to concatenated multimodal data, and different independent components (ICs) were identified as various aging patterns by clustering the mixing weights of those ICs. However, fusing multimodal features through decomposition methods such as ICA can be more challenging to explain the resulting features, which are linearly weighted combinations of original neuroimaging features. In summary, previous studies utilizing multimodal neuroimaging features to investigate aging have not yet elucidated how brain function and structure coevolve throughout the aging process as well as their impact on cognitive decline.

While previous multimodal age prediction studies have shown promising results, many used simple feature concatenation strategies that implicitly prioritized structural information, often at the expense of functional insights. This limits understanding of how brain structure and function co-evolve during aging. In contrast, our study introduces a more refined analytical framework that first independently evaluates the predictive contributions of each modality, and then assesses the added value of their joint features in prediction. Moreover, we go beyond prediction accuracy by conducting comprehensive analyses to examine joint aging patterns. This design allows us to offer a more nuanced and integrative understanding of the neurobiological aging process. In our work, a large dataset comprising 27,793 healthy subjects ranging from middle-aged to older adults is employed to maximize the reliability of our aging findings, overcoming the limited generalization ability of small sample studies [29,30,34,35] and improving the specificity in exploring aging relative to those using the lifetime datasets [25,30,35–38]. Furthermore, the association between joint brain changes and cognitive decline is evaluated to understand the complex relations among brain structure, function, and cognitive abilities during the aging process. Our work not only deepens the understanding of the neural basis of aging but also holds promise for the development of imaging-based biomarkers to detect early signs of age-related cognitive impairment.

## Results

**Fig. 1.**
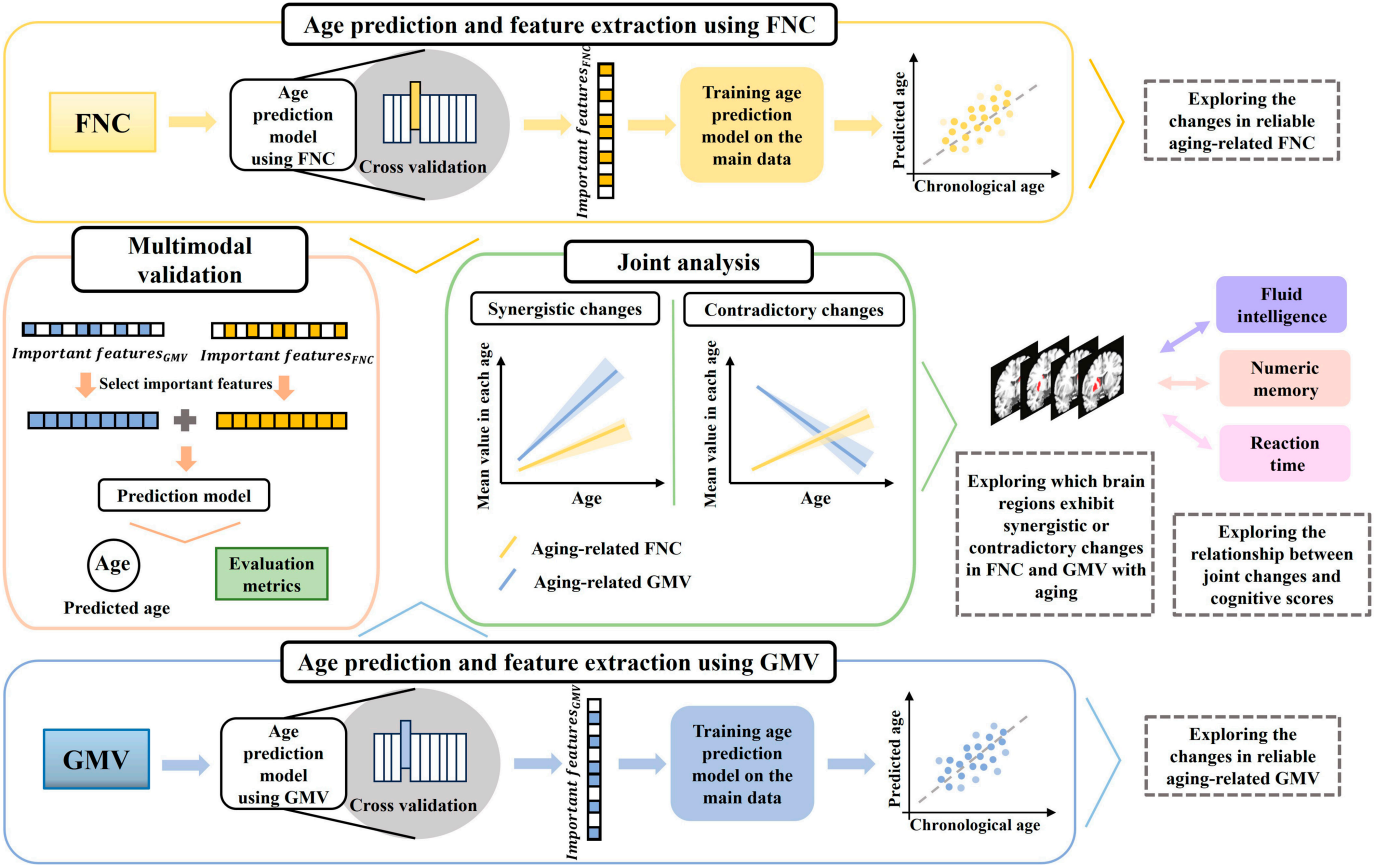
The overall analysis framework including both single-modal and multimodal brain age predictions followed by the exploration of separate changes in brain function and structure as well as the joint changes between brain function and structure with aging. First, single-modal age prediction models are trained separately to select important aging-related FNC and GMV features, and are then evaluated with respect to their age prediction abilities. Subsequently, those important FNC and GMV features are combined and validated by multimodal age prediction. Finally, in addition to investigating the separate changes in brain function and structure based on reliable aging-related FNC and GMV across multiple cross-validation repetitions, the interactions between functional and structural alterations during the aging as well as the associations between the joint function–structure changes and the cognitive declines are investigated.

Most subjects aged 49 to 76 years and having both functional network connectivity (FNC) and whole-brain GMV measures in the UK Biobank dataset were included for investigating complex aging effects on brain. The FNC was assessed by calculating partial correlations between the time series of any pair among 55 brain functional networks (shown in Fig. S1), resulting in 1,485 FNC features for each subject. For each network, we found its relevant brain regions (see Table S1) by matching with the combined Harford–Oxford and Diedrichsen cerebellar atlas [39]. Consistent with our previous work [40], those networks were assigned to 9 functional domains including the attentional (AT), auditory (AU), cerebellum (CB), cognitive control (CC), default mode (DM), frontoparietal (FP), subcortical (SC), sensorimotor (SM), and visual (VI) domains. The whole-brain GMV was obtained by computing the mean value across voxels in each of the 153 brain regions using the same brain atlas for each subject. As outlined in Fig. 1, an unbiased double-layer 10-fold cross-validation age prediction framework including both single-modal and multimodal predictions was applied to ensure the reliability of our findings. We first identified the most important aging-related FNC or GMV features within the inner cross-validation and then verified them using the independent data in the outer cross-validation. After that, we combined these important single-modal features to train additional multimodal age prediction models using the main data and then validated the robust association with aging of these multimodal features using the independent data. Furthermore, regarding the reliable overlapping multimodal features across multiple runs in the outer cross-validation procedure, we not only comprehensively investigated the unique alterations in brain function and structure but also proposed a novel multimodal joint analysis method to uncover the interplay mechanism between brain functional and structural changes while highlighting the concurrent evolution most closely associated with cognitive decline during the aging process.

### GMV demonstrates stronger predictive power than FNC, while multimodal data outperform single-modal data for age prediction

Our work first involved constructing single-modal age predictions using either FNC or GMV to explore and verify important changes in brain function or structure. We employed a nested 10-fold cross-validation framework for unbiased single-modal age prediction (see Methods). In the outer loop, the data were split into main and independent sets, balanced for age (49 to 76 years, 28 groups) and gender by random sampling. The inner loop further divided the main set into training and testing subsets. We used a least absolute shrinkage and selection operator (Lasso) regression to identify sparse, aging-related features from FNC or GMV, training models on the inner training data and selecting features based on performance on the inner test data. These features were then used to train a new Lasso model on the main data and evaluate prediction performance on the outer independent set. We further examined whether combining single-modal features could enhance age prediction. To ensure consistency, we used the same data partition and validation framework as in the single-modal analysis. For multimodal prediction, important FNC and GMV features were combined to train a new Lasso model on the main data and evaluated on the corresponding independent set. This yielded predicted brain ages for both single-modal (on inner testing and independent testing data) and multimodal models (on independent testing data).

**Fig. 2.**
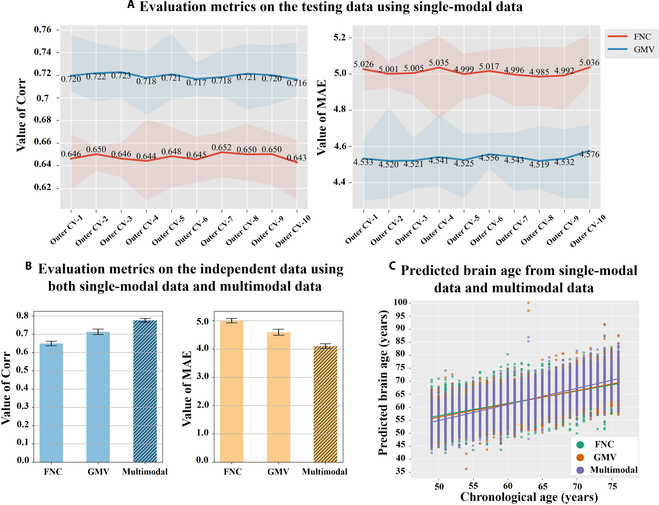
The performances of the age predictions using the single-modal and multimodal neuroimaging data. (A) Evaluation metrics on the testing data using the single-modal data. The *x* axis represents the outer cross-validation (CV) run, and the *y* axis denotes the Corr or MAE value. Each line displays the average Corr or MAE values using FNC or GMV across the testing datasets of the inner cross-validation along 10 outer CV runs, with shaded areas indicating the maximum and minimum of these Corr or MAE values. (B) Evaluation metrics for the FNC, GMV, and multimodal measures on the independent data of the outer cross-validation. Each error bar represents the average and standard deviation of Corr or MAE across the 10 independent datasets in the outer cross-validation. (C) Predicted brain ages using the single-modal data and multimodal data on the independent data. The *x* axis denotes the real chronological ages, and the *y* axis denotes the predicted brain ages using FNC, GMV, or the combined multimodal features.

Regarding the age prediction performance measured by Pearson correlation coefficient (Corr) and mean absolute error (MAE; unit: years) based on the subjects’ chronological ages and predicted brain ages, Fig. 2 illustrates the evaluation metrics on the testing data using FNC and GMV (Fig. 2A) and that on the independent data using both single-modal and multimodal features (Fig. 2B). Detailed prediction results are also included in Tables S2 and S3. Using the FNC features, we obtained an average Corr of 0.647 and an average MAE of 5.009 across the 100 testing datasets in the double-layer cross-validation, demonstrating a satisfied performance in the age prediction. Moreover, the average Corr and average MAE across 10 independent datasets in the outer cross-validation are 0.649 and 5.003, respectively, further reinforcing the reliability and generalizability of the FNC-based age prediction. Regarding the age prediction using GMV, our results indicate that the age prediction leveraging GMV resulted in better performance than that based on FNC, as GMV-based models yielded an average Corr of 0.719 and an average MAE of 4.537 across the testing data, and an average Corr of 0.713 and an average MAE of 4.596 across the independent data. These results align with prior knowledge that as people age, brain structural changes [31,41–43] are often more pronounced than functional alterations. Remarkably, as shown in Fig. 2B, the multimodal age prediction achieves an average Corr of 0.776 and an average MAE of 4.104 across the independent datasets, which is better than that in any single modality age prediction even though both single-modal and multimodal models are built using the same main datasets. In addition to the evaluation metrics, we show the scatter plots presenting the predicted brain ages and chronological ages using those features in Fig. 2C. Taken together, all the results support that combining brain functional and structural features offers substantial benefits in the age prediction, highlighting the importance of further exploring their interplay.

### Reliable aging-related FNCs and GMVs

**Fig. 3.**
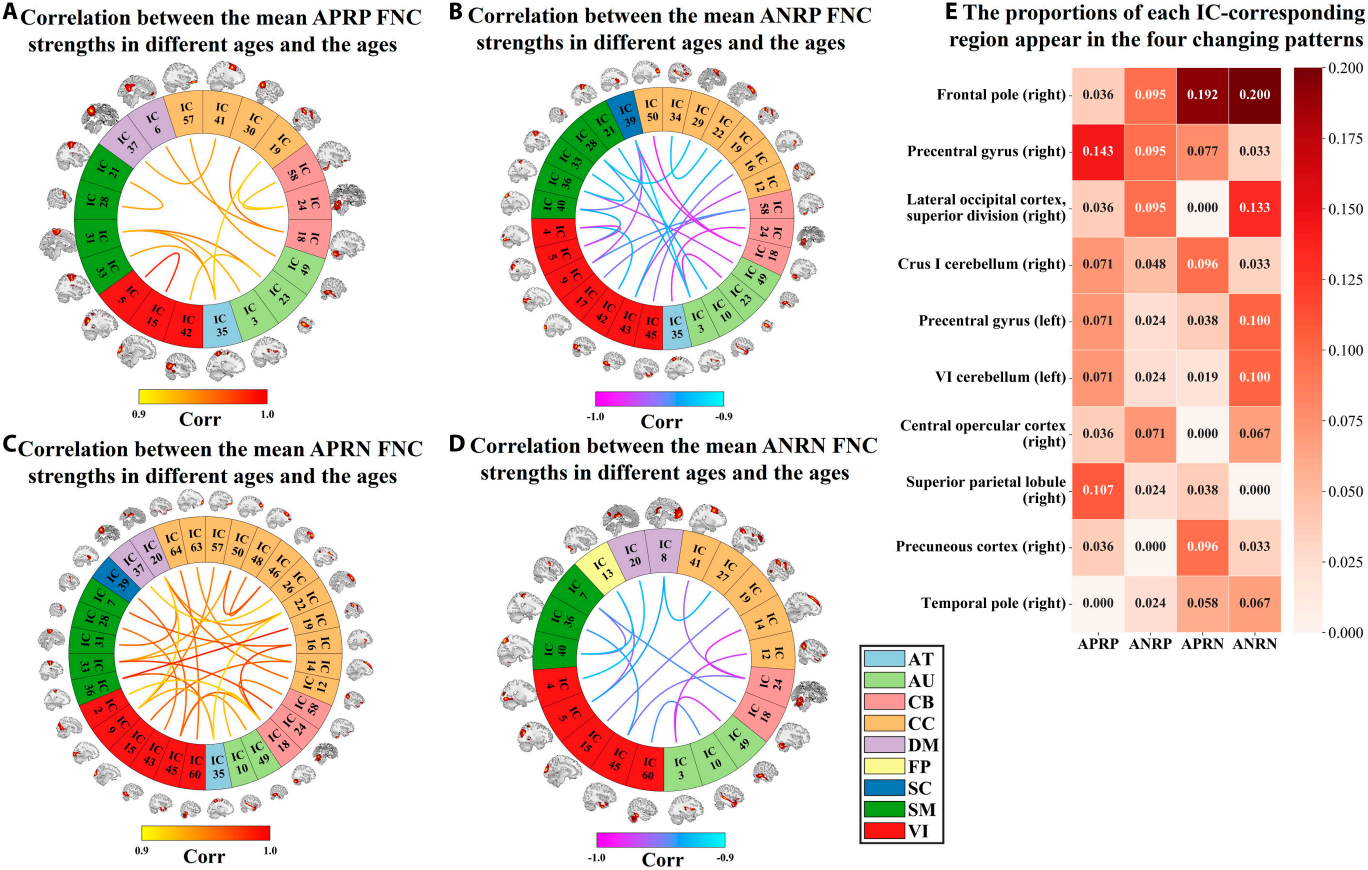
Seventy-seven reliable aging-related FNCs, each of which has an absolute correlation value greater than 0.9 to the chronological age. (A to D) FNC–age correlations for the FNCs that belong to 4 different changing patterns (APRP, ANRP, APRN, and ANRN). The FNC–age correlation was computed using the mean FNC strengths at different ages and the ages. The 55 networks (i.e., ICs) are divided into 9 domains including the attentional (AT), auditory (AU), cerebellum (CB), cognitive control (CC), default mode (DM), frontoparietal (FP), subcortical (SC), sensorimotor (SM), and visual (VI) domains. (E) Occurring proportion of each IC-corresponding region in each changing pattern. Here, we display the top 10 regions that have a greater proportion sum across the 4 patterns.

Given the effectiveness of the important FNC and GMV features in predicting age, we identified the intersection of the 10 sets of important single-modal features across 10 runs in the outer cross-validation procedure, pinpointing 219 reliable FNCs and 96 reliable GMVs for further in-depth investigation. Similar to our previous work [12], the reliable FNCs were divided into 4 changing patterns according to their strengths and Pearson correlation coefficients with age: age-positively-related positive (APRP) FNCs, age-negatively-related positive (ANRP) FNCs, age-positively-related negative (APRN) FNCs, and age-negatively-related negative (ANRN) FNCs (see Table S4). Since 77 of 219 reliable FNCs had greater correlations with age (absolute value > 0.9), Fig. 3A to D separately illustrate them in 4 changing patterns. It is observed that the identified aging-related FNCs are distributed across various functional domains, with a greater number of FNCs belonging to the ANRP and APRN patterns compared to other patterns. This suggests that brain functional interactions generally show a reduced magnitude as humans age. Subsequently, we summarized the proportions of different brain regions associated with these FNCs in each pattern to investigate what regions are mostly related to what specific changing patterns. As shown in Fig. 3E, the right frontal pole with the greatest proportion sum is mostly sensitive to aging. Among the regions corresponding to FNCs in the APRP pattern, the right precentral gyrus occupies a larger proportion compared to other regions, suggesting a notable interaction enhancement in this region along with the aging. Regarding the ANRP pattern, the right frontal pole, precentral gyrus, and lateral occipital cortex (superior division) show greater prominence, indicating reduced participant in information communication as the brain ages. For both APRN and ANRN patterns, the right frontal pole occupies a larger proportion compared to other regions, reflecting the complex changes in FNC associated in this region during aging.

**Fig. 4.**
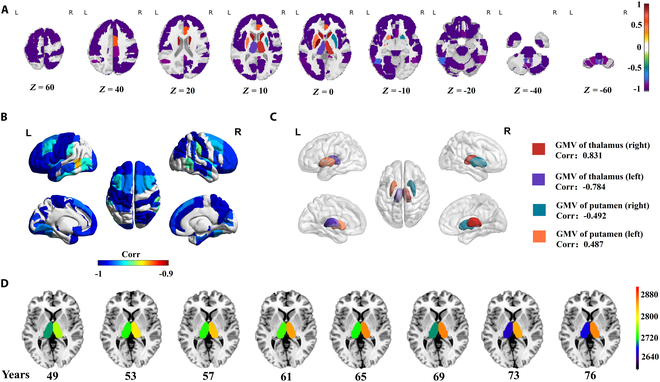
Correlation of reliable aging-related GMVs with chronological age. (A) Correlations between the GMV values at different ages and the chronological ages for all 96 reliable aging-related GMVs. (B) Strongly negative correlations (Corr < −0.9) between the GMV values at different ages and the ages for 74 of 96 reliable aging-related GMVs. (C) Opposite GMV–age correlations between the left and right thalamus as well as between the left and right putamen. (D) Mean GMV of the left and right thalamus at different ages.

Because GMV always represents positive values, all 96 reliable aging-related GMV features were divided into 2 changing patterns: age-positively-related (AP) GMV and age-negatively-related (AN) GMV (see Table S5). Figure 4A demonstrates that most GMVs in the brain belong to the AN pattern and tend to symmetrically decrease, although a few regions (e.g., the left and right caudate) also show increased GMV with aging. Strong negative correlations (correlation < −0.9) constitute 74 of 96 regions (Fig. 4B), with the top 4 significant negative correlations found in the GMV of the left precentral gyrus (correlation: −0.995), the right paracingulate gyrus (correlation: −0.995), and the left and right postcentral gyrus (correlation: −0.995 and −0.995). Interestingly, the GMVs in some regions on the left and right hemispheres exhibit opposite aging trends (Fig. 4C), suggesting possible lateralization during brain aging. Specifically, the GMVs in the left and right thalamus negatively and positively correlate with chronological ages (correlation: −0.784 versus 0.831), respectively, while the GMVs in the left and right putamen are positively and negatively correlated with chronological ages (correlation: 0.487 versus −0.492), respectively. Examining the thalamus, it is noteworthy that at age 49, the mean GMV values of the left and right thalamus are quite similar. However, as age increases, their trajectories of change diverge greatly, and the left thalamus exhibits a trend of fluctuating decrease in GMV, while its corresponding right region increases. Although previous work has found that the asymmetry of the putamen is varied with age [44] and the asymmetry of the thalamus is significant in younger groups [45], our study more precisely revealed opposite changing trends of GMV in the putamen and thalamus with aging.

### Joint changes between FNCs and GMVs during the aging process

**Fig. 5.**
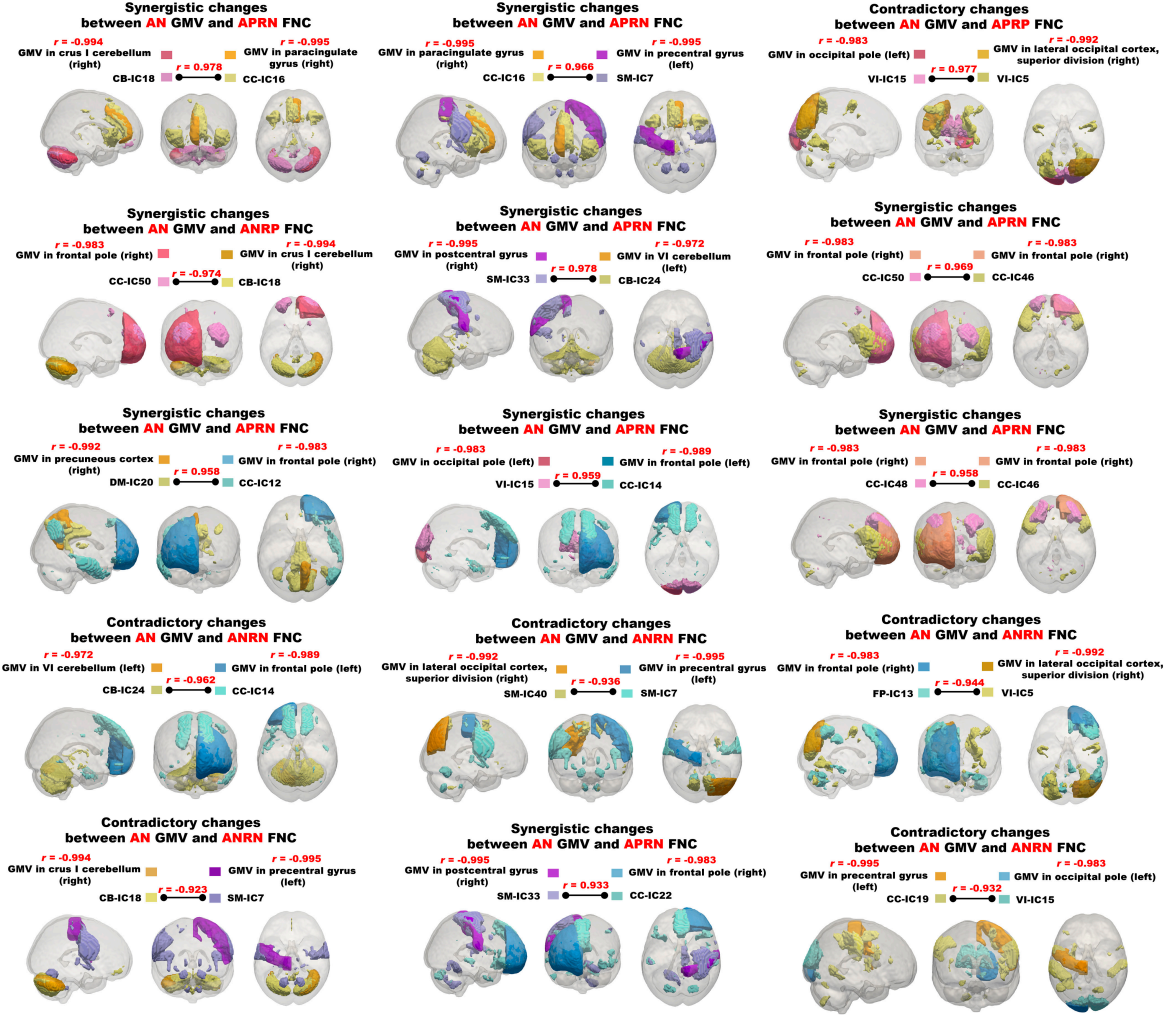
Synergistic and contradictory joint changes between FNC and GMV. Each subfigure shows one joint FNC-GMV change. For each joint FNC-GMV change, we not only display the brain regions for the FNC and its 2 corresponding GMVs but also include the neuroimage–age correlations (*r* values) for the FNC and the GMVs.

Based on the reliable aging-related features, we further investigated the joint changes between FNCs and GMVs, focusing on their potentially synergistic or contradictory aging trajectory. Specifically, we matched each FNC with its linked 2 brain regions to obtain the 2 corresponding GMVs, resulting in 96 joint FNC-GMV changes. Synergistic changes mean that FNC and its 2 corresponding GMVs all show consistent changing trends with both FNC and GMV decreased or increased along with aging, while contradictory changes indicate that FNC exhibits an opposite changing trend compared to 2 GMVs. We measured each joint FNC-GMV change using the 3 neuroimage–age correlations. Subsequently, we conducted a detailed analysis specifically for the top 15 joint FNC-GMV changes that exhibited stronger joint correlations (≥0.97) with age (Fig. 5). We found that synergistic joint changes (i.e., 9 of 15 changes) were predominant, involving both FNC and GMV decreased primarily in the cerebellum, frontal pole, paracingulate gyrus, and precuneus cortex of the CB, CC, and DM domains, indicating their concurrent reduction in GMVs and FNCs. Contradictory joint changes mainly occur in the occipital pole and lateral occipital cortex (superior division) in the VI domain as well as the frontal pole in the FP domain, which present increased FNC magnitude but decreased GMVs in the 2 corresponding brain regions. The results support a compensatory effect in these FNCs to offset the typical reduction of GMVs during aging. In addition, the left precentral gyrus in the SM domain demonstrates complex joint FNC-GMV changes, encompassing both synergistic and contradictory changes. In short, we provide evidence of functional degeneration occurring alongside structural changes, while some functional connectivity may exhibit compensatory responses to counteract the reduction in brain structure in certain brain regions.

### Relationship between joint FNC-GMV changes and cognitive declines

To explore the intricate relationship between joint FNC-GMV changes and cognitive declines, we carefully investigated the Pearson correlations between neuroimaging features indicative of significant joint changes and various cognitive scores including fluid intelligence (FI), numeric memory (NM), and reaction time (RT). The correlation results are illustrated in Table S6. It is observed that the joint change most strongly associated with both FI and NM involved contradictory FNC-GMV changes, characterized by increased FNC between the left occipital pole and the right lateral occipital cortex (superior division), accompanied by decreased GMVs in these regions. This finding suggests that regions within the visual domain play a critical role in modulating FI and NM, potentially by enhancing functional interaction to compensate for structural decline. In contrast, the joint change most strongly associated with RT showed concurrent decreases in both FNC and GMV between the right crus I of the cerebellum (CB domain) and the right paracingulate gyrus (CC domain). This result indicates that age-related declines in both structural and functional integrity in these regions may directly contribute to slower cognitive reaction speed.

**Fig. 6.**
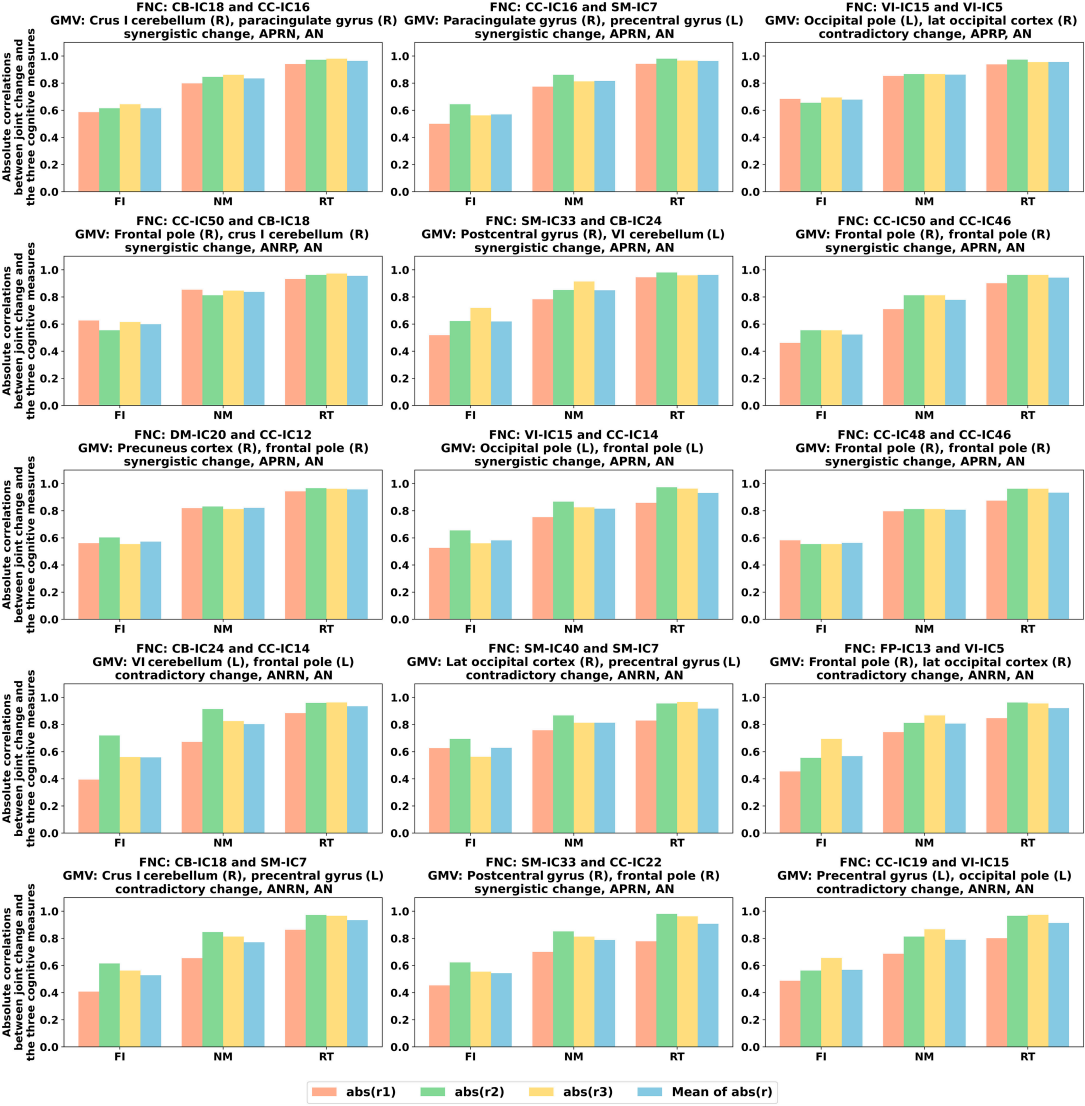
Absolute correlations between each joint change and the 3 cognitive measures examined in this study: FI, NM, and RT. The joint changes shown here are the same as those presented in Fig. 5. “r1” represents the correlation between the mean values of the FNC at different ages and the mean values of each cognitive score at different ages. “r2” represents the correlation between the mean values of GMV1 at different ages and the mean values of each cognitive score at different ages. “r3” represents the correlation between the mean values of the GMV2 at different ages and the mean values of each cognitive score at different ages. The 2 GMVs are associated with the FNC after matching them according to the same atlas. abs(r1), abs(r2), and abs(r3) denote the absolute values of r1, r2, and r3, respectively. “Mean of abs(r)” represents the mean of the absolute values of the 3 correlations (r1, r2, and r3).

In addition, as shown in Fig. 6, RT and NM exhibit stronger associations with joint changes compared to FI, based on the absolute values of Pearson correlation coefficients (original values reported in Table S6). These results are consistent with prior knowledge that FI tends to be more resilient to aging effects, while RT and memory-related functions are more vulnerable. This variability in aging sensitivity among the cognitive measures enhances the robustness of our findings, as it allows us to examine how brain features relate to cognitive domains with differing aging trajectories.

## Discussion

In the study, we aim to investigate the alterations in the brain during the normal aging process, particularly the joint changes between brain function and structure as well as their impacts on cognitive declines. We propose an effective framework that includes both single-modal and multimodal age predictions with a nested cross-validation to ensure the identification of key multimodal neuroimaging biomarkers associated with aging. Using a large dataset comprising 27,793 healthy adults, we carefully balance the sample sizes across different age groups as well as between genders. This approach is employed to minimize potential biases and confounding effects, thereby enhancing the reliability and validity of our age prediction results. In addition, we not only identify reliable aging-related FNC and GMV, respectively, but also uncover the significant joint brain FNC-GMV changes and their unique associations with different cognition declines.

Using single-modal Lasso models, we demonstrate that the chronological ages of individual subjects can be accurately predicted using either FNC or GMV. Remarkably, GMV outperforms FNC in terms of the age prediction accuracy, indicating that structural changes in the brain due to aging are more pronounced than functional changes, consistent with existing knowledge [31,34,46,47]. Regarding the reliable aging-related FNCs, most of them show decreased connectivity magnitudes during aging, highlighting the reduced neural synchronization, which is supported by many previous studies [21,48–51]. For instance, our previous work [12] using a different brain parcellation to investigate FNC also observed the hypoconnectivity trend in aging. Notably, our current study reveals that FNCs associated with the frontal pole exhibit the most significant and diverse changes with aging, highlighting their complexity in the aging brain compared to the typical pattern of hypoconnectivity. It has been acknowledged that frontal pole plays an important role in maintaining human behavior and cognitive abilities [52–54] but its subregions’ connectivity can be complicated [52]. Our work shows that the frontal pole exhibits weakened magnitude in negative FNCs mainly linked with the precuneus cortex and postcentral gyrus regions, likely resulting from functional reorganization during aging. The frontal pole primarily oversees complex cognitive functions such as decision-making, executive control, and adaptation to changing environments [55]. The precuneus cortex involves episodic memory retrieval and self-related cognitive activities [56]. Meanwhile, the postcentral gyrus is responsible for general sensations of touch, pressure, proprioception, and so on [57]. The diminished negative connectivity may suggest an impairment of the inhibition about self-perception during tasks such as decision-making. In contrast, the frontal pole presents an enhanced communication attempt in negative FNCs, primarily with the lateral occipital cortex, potentially serving as a maintenance of brain function between complex cognition tasks and visual processing in the elderly [58,59]. Moreover, the right precentral gyrus demonstrates enhanced amplitude in positive FNCs primarily linked with postcentral gyrus, central opercular cortex, planum temporale, and superior parietal lobule as aging progresses. Indeed, the precentral gyrus primarily controls and executes voluntary movements [60], and its enhanced interaction with other brain areas may be a well-maintained mechanism in response to a decline in motor control and coordination. In terms of the reliable aging-related GMVs, most changes occur in a symmetrical manner with reduced GMV, reflecting a general loss of neurons in neuronal density that could be linked to decline in cognitive abilities, which accords with previous studies [61,62]. Particularly, our study reveals for the first time that both the left and right caudate show increased GMV with aging. The caudate is essential for a variety of functions, including motor control, cognitive processes, and emotional regulation [63,64]. This increase of neural density may contribute to preserving these core functions and adapting to changes over time. More interestingly, a notable asymmetry pattern is found in the GMV alterations of specific regions as the brain ages, especially in the thalamus and putamen. Although earlier studies have demonstrated that the asymmetry of the putamen changes with age [44], and the asymmetry of the thalamus is significant in younger populations [45], our research provides more precise evidence that aging has specific laterality on the regions.

As expected, our study illustrates that multimodal age prediction combining FNC and GMV consistently outperforms that using single-modal features, similar to other multimodal studies [34,65]. This supports a growing consensus that age-related brain changes are multifactorial and interconnected, rather than localized or isolated to a single modality. We further show the importance of studying aging mechanism from both function and structure, especially their joint aging path. Both synergistic and contradictory changes between FNC and GMV are found in our study. In particular, the cerebellum, frontal pole, paracingulate gyrus, and precuneus cortex exhibit synergistic changes characterized by decreased FNC and GMV with advancing age, indicating simultaneous functional and structural degeneration in motor control, sensory processing, and emotional regulation in elderly individuals. Previous studies have shown that functions related to sensorimotor integration and cognitive control typically decline as part of the natural aging process [66–68]. Contradictory changes are observed in regions such as the occipital pole and lateral occipital cortex (superior division), related to visual functions, as well as the frontal pole, associated with cognitive and motor functions. These changes of increased FNC alongside decreased GMV in these regions may act as a compensatory mechanism to counteract the loss of GMV to preserve visual acuity, cognitive ability, and behavioral control in the elderly [69,70]. Taken together, our findings underscore that natural brain aging involves both concurrent degeneration and adaptive balancing mechanisms in brain function and structure.

Our findings disclose marked correlations between joint FNC-GMV changes and cognitive scores, especially with RT. Notably, the contradictory FNC-GMV changes related to FI and NM are primarily observed in the brain’s visual information processing areas, such as the occipital pole and the lateral occipital cortex. As age progresses, the visual-related regions undergo potential compensatory changes in function and structure to maintain and enhance the visual precision and memory capabilities of the elderly. Previous studies have demonstrated that while structural decline occurs, functional reorganization may take place, such as increased local or network-level activity, to counteract these changes and preserve essential cognitive functions like visual imagery and working memory [71]. The prominence of these effects in the visual modality, as opposed to other sensory systems, may be due to the visual system’s extensive cortical representation and its integral role in higher-order cognitive processing. Furthermore, the synergistic FNC-GMV decreases related to the crus I cerebellum and the paracingulate gyrus exhibit a strong association with the RT. The crus I cerebellum, contributing to working memory [72], and the paracingulate gyrus, crucial for attention [73], likely deteriorate jointly over the course of aging, which may significantly slow RT. In summary, our study discloses that specific age-related joint changes critically impair unique aspects of cognitive function.

In summary, we found that synergistic declines are predominant, while contradictory changes may reflect compensatory mechanisms to counter structural loss. First, we identified a subset of regions with synergistic GMV-FNC changes, suggesting that these structural–functional changes are particularly vulnerable to age-related degeneration. Notably, the cerebellar–frontal circuits demonstrated such coupled decline, with both structural atrophy and functional weakening closely associated with impairments in motor control, emotional regulation, and executive functions. This pattern aligns with previous evidence indicating the central role of these regions in integrating cognitive and motor processes during aging [74,75]. Second, we observed regions where GMV decreased while FNC increased, consistent with compensatory functional reorganization. This finding supports the view that the aging brain may dynamically reorganize functional networks to partially offset the impact of structural decline [76]. Such functional enhancement occurred not only between homotopic regions but also across distinct networks, potentially serving to maintain cognitive performance. Moreover, our cognitive–neuroimaging analyses reveal notable contradictory FNC–GMV changes associated with FI and NM, suggesting that compensatory neural hyperactivity may arise to counteract structural deterioration and help preserve these functions, consistent with recent neuroimaging findings [77]. These findings collectively emphasize that brain aging is not a unidirectional degenerative process but rather involves dynamic adjustments and reconfigurations across specific networks and hemispheres, aimed at preserving functional stability and cognitive capacity for as long as possible.

There are a few limitations that may be addressed in future studies. First, while we utilized the UK Biobank dataset—a well-established, large-scale, and high-quality resource with extensive validation—the generalizability of our aging findings could be further assessed in more diverse and representative populations. Second, we did not adjust for educational attainment due to the heterogeneous and incomplete recording of education levels in the UK Biobank. While the distribution of education appears relatively balanced across age groups in our sample (Table S7), the lack of standardized data prevents formal adjustment. Third, we did not consider the gap between the brain age and the chronological age, and directly used the chronological age for building the age prediction models. Addressing this gap beforehand could potentially improve the accuracy of age prediction models [78,79], although it remains challenging to account for this discrepancy accurately. Fourth, in terms of the identified reliable aging-related features, our study primarily focused on the linear relationship between neuroimaging measures and age (or specific cognitive score). Studies have shown that the aging brain may exhibit nonlinear characteristics [80]; therefore, future studies can be conducted to explore these nonlinear relationships to gain a deeper understanding of their complexity. Fifth, to maximize the sample size, we used cross-sectional data to study aging, similar to many existing work [81,82]. In the future, longitudinal studies may enhance the validity of our findings. Lastly, we used the Lasso model for age prediction, prioritizing its feature interpretability. However, deep learning models [83] incorporated with feature explanation capabilities could also be used to study joint changes and may improve the age prediction performance.

In summary, utilizing effective and unbiased age predictions followed by an innovative multimodal joint analysis, our study discovers the impact of normal aging on the intricate interplay between brain function and structure across the entire brain. We demonstrate the superior capability of multimodal measures in age prediction. Furthermore, our exploration of joint FNC-GMV changes reveals that synergistic declines dominate, with functions potentially compensating for structural alterations within contradictory changes, offering profound insights into the comprehension of the aging process. Additionally, our findings indicate a significant strong association between specific joint FNC-GMV changes and the declines in distinct cognitive abilities. Overall, our work underscores the critical importance of examining joint functional and structural changes during aging, offering a novel perspective for deepening our understanding of the brain’s aging process. By elucidating the interplay between structural and functional alterations, our findings may inform intervention strategies that target specific structure–function couplings to preserve cognitive health. This contributes to ongoing efforts in aging and cognitive neuroscience to advance more personalized and preventative approaches to brain health monitoring.

## Methods

### Large-sample datasets with both brain functional and structural measures

Based on 40,771 subjects that have the FNC measures and 42,794 subjects that have the GMV measures in the UK Biobank project, we selected healthy subjects who are aged 49 to 76 and have both measures for our study, as our primary goal was to disclose the joint changes between brain function and structure during aging. In order to investigate the normal aging mechanism, the subjects diagnosed with any mental, neural system, or other diseases that could potentially affect the brain, based on the criteria defined in the International Classification of Diseases tenth version (ICD-10), were discarded. To reduce the effect of head motion, we further excluded the subjects with head movements exceeding 0.5 mm while scanning. Following these criteria, we obtained a large dataset including 27,793 subjects (14,862 females and 12,931 males) for exploring the aging path in terms of brain function and structure.

The brain functional and structural measures were downloaded from the UK Biobank website. The used brain functional measures were the functional connectivity among 55 brain functional networks, referred to as FNC, given that there has been strong evidence that brain function is greatly impacted when people age, thus affecting memory, learning, and other cognitive functions [84,85]. These networks were obtained by performing a group ICA [86,87] on the denoised fMRI data by the UK Biobank team and assigned into 9 functional domains in our work. For each network, we also matched it to the brain regions of the combined brain atlas using the Harford–Oxford atlas and Diedrichsen cerebellar atlas by our developed intelligent analysis of brain connectivity (IABC) toolbox [88], thus identifying its 3 most relevant brain regions. For each subject, a 55×55 FNC matrix was calculated using Z-scored partial correlations between any 2 networks’ time series. Due to the symmetricity of each FNC matrix, only its lower triangular elements were used as the FNC measures (size: 1 ×1,485) for investigating aging-related brain functional changes. For brain structural measures, the whole brain’s GMV values were employed to investigate the impact of brain aging on brain structure. Based on the same brain atlas, the GMV values of all voxels in each brain region were averaged to represent the GMV in the region, thus resulting in 153 GMV measures for each subject.

### A unified framework including both single-modal and multimodal age predictions for exploring brain functional and structural changes along aging

Since Lasso regression typically shows a better performance compared to other regression approaches due to its ability in selecting sparse features for more effective prediction in the neuroscience field [46,89–91], we utilized Lasso with an L1-norm penalty for both the single-modal (using FNC or GMV) and subsequent multimodal age predictions in this study. As outlined in Fig. S2A, for the single-modal age prediction, we used a double-layer 10-fold cross-validation to maximize the reliability of extracting important aging-related brain changes in function or structure as well as to guarantee the unbiasedness of verifying their prediction performance using the independent data. To address the variability in the number of subjects between females and males and across 28 age groups (ranging from 49 to 76 years), we implemented a random sampling strategy designed to ensure a consistent and balanced sample size across all age groups. Specifically, we standardized the number of participants to 322 per age group. Within each age group, an equal number of females and males—166 each—were randomly selected for every outer cross-validation run. This balanced sampling approach minimizes potential biases arising from unequal group sizes or gender imbalances, thereby improving the robustness and generalizability of our analyses. After that, regarding each outer 10-fold cross-validation procedure, we divided the sampled subjects into the main and independent testing datasets (9:1). The main dataset was then further partitioned into 10 folds for the inner cross-validation by employing 9 folds of training data for building the Lasso model and the remaining fold of inner testing data for evaluating its prediction performance. Since greater prediction accuracy means a more effective model constructed using age-relevant features, we identified the important sparse neuroimaging features (FNC or GMV) that corresponded to the model with the highest accuracy on the testing data within the inner cross-validation. Next, the features were further utilized for training additional Lasso model using the main data and examined using the independent testing data in the outer cross-validation. Therefore, we obtained 10 sets of important features as well as the predicted brain ages on both the inner testing and outer independent testing data for the single-modal age prediction using FNC or GMV. In our work, we computed the Corr and MAE between the subjects’ chronological ages and predicted ages for evaluating the performance accuracy of age prediction.

Following the identification of aging-related FNC or GMV using the single-modal age predictions, we combined the selected important features from both modalities and built additional Lasso model using the entire main data and then tested its performance on the independent testing data in the outer cross-validation. Consequently, for the multimodal age prediction, we obtained metrics of prediction accuracy for 10 independent datasets in the outer cross-validation procedure. It is expected that the multimodal prediction would yield better performance than the single-modal predictions, thus supporting the importance of brain changes in both function and structure along aging.

### Investigation of brain functional and structural changes and their joint brain aging path

As mentioned above, 10 sets of important FNC or GMV features were obtained through the double-layer 10-fold cross-validation. We took their overlapping features as the most reliable aging-related FNC or GMV features and then comprehensively analyzed their properties. For each reliable aging-related FNC, we calculated the mean connectivity strength across all subjects for each age group and then computed the Pearson correlation between the mean FNC strengths at different ages and the chronological ages. Each mean FNC strength was calculated across subjects for each age group. Next, consistent with our previous work [12], these FNCs were assigned into 4 groups with different changing patterns (i.e., APRP, ANRP, APRN, and ANRN FNCs) to disclose what FNCs show what specific patterns. Taking the APRP pattern for instance, the FNC belonging to this pattern has a mean positive connectivity strength across all subjects and shows a positive correlation between its mean FNC strengths at different ages and ages. Since we obtained the 3 most relevant regions for each functional network (i.e., IC) by matching the IC to the brain atlas, the most relevant region was taken as the IC-corresponding region. For each changing pattern (e.g., APRP), we calculated the occurring proportion of each IC-corresponding region within all regions that relate to FNCs belonging to the pattern. After that, we summed the proportions of each IC-corresponding region appearing in the 4 changing patterns and ranked those regions according to the total proportion. For investigating the reliable aging-related GMVs, the GMVs were assigned to 2 changing patterns (i.e., AP GMV and AN GMV) based on their correlations between the mean GMV values at different ages and the chronological ages. As such, an AP GMV has a positive correlation and an AN GMV has a negative correlation between its mean GMV values at different ages and the chronological ages. In the computation, the mean GMV across all subjects in each age group was used to represent the GMV in the age.

In addition to investigating the brain’s functional and structural changes separately, a more important goal of our study is to explore the joint brain function–structure aging path. Based on the obtained reliable aging-related FNCs and GMVs, we explored joint FNC-GMV changes by matching each FNC with the most relevant linked brain regions according to Table S1 to find its 2 corresponding GMVs. Regarding each joint FNC-GMV change, we were interested in exploring if the FNC changes along with 2 corresponding GMVs in a synergistic or contradictory manner. We regarded the joint changes where both the GMVs’ values and the FNC’s magnitude consistently increase or decrease with age as synergistic changes; otherwise, they are considered contradictory changes. Here, we measured the joint FNC-GMV changes along with aging based on the neuroimage–age correlations from the involved FNC and corresponding GMVs using Eqs. (1) to (4). The value of *r* represents the degree of joint change. We have designed a unique calculation method for the joint change associated with each FNC changing pattern. Take the $r_{\text{APRP}\;\mathrm{FNC}-\mathrm{GMV}}$ as example, where APRP FNC refers to an FNC with the APRP pattern, if its 2 corresponding GMVs are both positively correlated with age, it indicates a synergistic joint change; on the contrary, if its 2 corresponding GMVs are both negatively correlated with age, it signifies a contradictory joint change.

rAPRPFNC−GMV=rFNC+rGMV1+rGMV23,rGMV>0Synergistic changerFNC−rGMV1−rGMV23,rGMV<0Contradictory change (1)

rANRPFNC−GMV=−rFNC−rGMV1−rGMV23,rGMV<0Synergistic change−rFNC+rGMV1+rGMV23,rGMV>0Contradictory change (2)

rAPRNFNC−GMV=rFNC−rGMV1−rGMV23,rGMV<0Synergistic changerFNC+rGMV1+rGMV23,rGMV>0Contradictory change (3)

rANRNFNC−GMV=−rFNC+rGMV1+rGMV23,rGMV>0Synergistic change−rFNC−rGMV1−rGMV23,rGMV<0Contradictory change (4)

Here, $r_{\mathrm{FNC}}$ represents the correlation between the mean FNC strengths at different ages and ages, and $r_{\mathrm{GMV}}$ represents the correlation between the mean GMV values at different ages and ages. The mean neuroimaging measure was computed based on all subjects in each age group.

### Quantitative analysis of the associations between joint brain function–structure changes and cognitive declines

Since aging people tend to present impaired cognitive abilities in memory, learning, and reaction, we further investigated the associations between the joint FNC-GMV changes and cognitive declines. In this study, the cognitive scores of interests included the commonly used FI, NM, and RT. Since the UK Biobank dataset lacks a standardized full-scale intelligence quotient (IQ) measure, the FI score—widely used in large-scale neuroimaging and cognitive studies—was adopted in this study as a proxy for general intelligence. For each age group, we calculated the mean feature value for each FNC and its 2 corresponding GMVs for the significant joint changes, as well as the mean cognitive score across all subjects within the age group. Next, we calculated the correlation between the mean values of each neuroimaging feature (FNC and 2 GMVs) at different ages and the mean values of each cognitive score (FI, NM, and RT) at different ages. For each significant joint change, we have identified a total of 9 correlations. For each cognitive score, we calculated the mean of the absolute values of the 3 correlations. Through this method, we have ultimately determined the joint change that is most strongly associated with each cognitive score.

## Data Availability

We used data from the UK Biobank datasets with the agreement of project 34175 (principal investigator: Y.D.) that do not allow for redistribution; however, the UK Biobank data can be accessed directly from the UK Biobank repository. All codes for the data analysis will be available.
