# Supplementary material for: Joint Aging Patterns in Brain Function and Structure Revealed Using 27,793 Samples
Source: Research (Wash D C). 2025 Sep 25;8:0887. doi: 10.34133/research.0887 (PMC12460996; doi:10.34133/research.0887)
Supplement: Supplementary 1 — Tables S1 to S7 Figs. S1 and S2 [file research.0887.f1.docx]

Supplemental information

**Table S1. Information of 55 brain functional networks.** For each network, we include its functional domain, IC ID, and the most related three brain regions in the combined Harford-Oxford and Diedrichsen cerebellar atlas.

| **Domain** | **IC ID** | **Brain region (voxel percentage in the network)** |
| --- | --- | --- |
| AT | IC 35 | Superior Parietal Lobule (right) (0.106), Superior Parietal Lobule (left) (0.099), Postcentral Gyrus (left) (0.068) |
|  | IC 52 | Middle Temporal Gyrus, temporooccipital part (right) (0.144), Middle Temporal Gyrus, temporooccipital part (left) (0.121), Cingulate Gyrus, posterior division (right) (0.071) |
| AU | IC 3 | Central Opercular Cortex (right) (0.057), Insular Cortex (left) (0.055), Central Opercular Cortex (left) (0.055) |
|  | IC 10 | Temporal Pole (left) (0.112), Temporal Pole (right) (0.099), Middle Temporal Gyrus, posterior division (left) (0.052) |
|  | IC 23 | Planum Temporale (left) (0.053), Planum Temporale (right) (0.042), Superior Temporal Gyrus, posterior division (right) (0.038) |
|  | IC 49 | Middle Temporal Gyrus, posterior division (left) (0.185), Middle Temporal Gyrus, posterior division (right) (0.151), Inferior Temporal Gyrus, posterior division (left) (0.07) |
| CB | IC 18 | Crus I Cerebellum (right) (0.216), Crus I Cerebellum (left) (0.163), Crus II Cerebellum (right) (0.135) |
|  | IC 24 | VI Cerebellum (left) (0.081), VI Cerebellum (right) (0.078), Crus I Cerebellum (right) (0.053) |
|  | IC 58 | VIIIa Cerebellum (right) (0.21), Inferior Temporal Gyrus, temporooccipital part (right) (0.204), VIIb Cerebellum (right) (0.078) |
| CC | IC 12 | Frontal Pole (right) (0.128), Angular Gyrus (right) (0.109), Lateral Occipital Cortex, superior division (right) (0.106) |
|  | IC 14 | Frontal Pole (left) (0.163), Frontal Pole (right) (0.146), Superior Frontal Gyrus (left) (0.112) |
|  | IC 16 | Paracingulate Gyrus (right) (0.097), Paracingulate Gyrus (left) (0.087), Cingulate Gyrus, anterior division (right) (0.082) |
|  | IC 19 | Precentral Gyrus (left) (0.081), Supramarginal Gyrus, anterior division (left) (0.073), Postcentral Gyrus (left) (0.07) |
|  | IC 22 | Frontal Pole (right) (0.1), Superior Frontal Gyrus (right) (0.083), Superior Frontal Gyrus (left) (0.073) |
|  | IC 26 | Middle Frontal Gyrus (left) (0.088), Middle Frontal Gyrus (right) (0.079), Frontal Pole (right) (0.053) |
|  | IC 27 | Frontal Pole (right) (0.104), Lateral Occipital Cortex, superior division (right) (0.101), Middle Frontal Gyrus (right) (0.056) |
|  | IC 29 | Superior Frontal Gyrus (left) (0.107), Inferior Frontal Gyrus, pars opercularis (left) (0.081), Crus I Cerebellum (right) (0.071) |
|  | IC 30 | Angular Gyrus (right) (0.093), Supramarginal Gyrus, posterior division (left) (0.081), Supramarginal Gyrus, posterior division (right) (0.078) |
|  | IC 32 | Frontal Pole (left) (0.244), Frontal Pole (right) (0.149), Middle Frontal Gyrus (left) (0.137) |
|  | IC 34 | Frontal Pole (right) (0.105), Superior Frontal Gyrus (right) (0.072), Crus I Cerebellum (left) (0.046) |
|  | IC 38 | Frontal Pole (right) (0.204), Frontal Pole (left) (0.177), Middle Frontal Gyrus (left) (0.128) |
|  | IC 41 | Superior Frontal Gyrus (left) (0.157), Superior Frontal Gyrus (right) (0.138), Middle Frontal Gyrus (right) (0.105) |
|  | IC 46 | Frontal Pole (right) (0.154), Frontal Pole (left) (0.134), Inferior Frontal Gyrus, pars opercularis (left) (0.067) |
|  | IC 48 | Frontal Pole (right) (0.209), Frontal Pole (left) (0.186), Middle Frontal Gyrus (left) (0.099) |
|  | IC 50 | Frontal Pole (right) (0.381), Frontal Pole (left) (0.305), Superior Frontal Gyrus (right) (0.047) |
|  | IC 57 | Frontal Pole (right) (0.205), Frontal Pole (left) (0.169), Frontal Orbital Cortex (right) (0.079) |
|  | IC 63 | Frontal Pole (right) (0.333), Frontal Pole (left) (0.321), Frontal Orbital Cortex (right) (0.026) |
|  | IC 64 | Frontal Pole (left) (0.063), Crus I Cerebellum (right) (0.056), Middle Temporal Gyrus, posterior division (left) (0.05) |
|  | IC 93 | Frontal Pole (right) (0.129), Frontal Pole (left) (0.098), Brain-Stem (0.014) |
| DM | IC 6 | Precuneus Cortex (left) (0.291), Lateral Occipital Cortex, superior division (left) (0.283), Cingulate Gyrus, posterior division (left) (0.184) |
|  | IC 8 | Frontal Pole (right) (0.092), Frontal Pole (left) (0.091), Paracingulate Gyrus (right) (0.06) |
|  | IC 11 | Lateral Occipital Cortex, superior division (left) (0.142), Lateral Occipital Cortex, superior division (right) (0.113), Precuneus Cortex (right) (0.104) |
|  | IC 20 | Precuneus Cortex (right) (0.144), Precuneus Cortex (left) (0.117), Cingulate Gyrus, posterior division (right) (0.103) |
|  | IC 37 | Precuneus Cortex (right) (0.322), Precuneus Cortex (left) (0.219), Cingulate Gyrus, posterior division (right) (0.077) |
|  | IC 53 | Lateral Occipital Cortex, superior division (left) (0.182), Angular Gyrus (left) (0.105), Angular Gyrus (right) (0.101) |
| FP | IC 13 | Frontal Pole (right) (0.217), Middle Frontal Gyrus (right) (0.122), Crus I Cerebellum (left) (0.09) |
|  | IC 25 | Lateral Occipital Cortex, superior division (left) (0.18), Lateral Occipital Cortex, superior division (right) (0.126), Middle Frontal Gyrus (left) (0.076) |
| SC | IC 39 | Putamen (left) (0.139), Putamen (right) (0.134), Caudate (left) (0.087) |
| SM | IC 7 | Precentral Gyrus (left) (0.117), Postcentral Gyrus (left) (0.117), Precentral Gyrus (right) (0.109) |
|  | IC 21 | Postcentral Gyrus (left) (0.187), Precentral Gyrus (left) (0.158), Superior Parietal Lobule (left) (0.076) |
|  | IC 28 | Precentral Gyrus (right) (0.183), Precentral Gyrus (left) (0.181), Superior Frontal Gyrus (right) (0.064) |
|  | IC 31 | Precentral Gyrus (right) (0.115), Postcentral Gyrus (left) (0.109), Precentral Gyrus (left) (0.108) |
|  | IC 33 | Postcentral Gyrus (right) (0.215), Precentral Gyrus (right) (0.189), Superior Parietal Lobule (right) (0.077) |
|  | IC 36 | Precentral Gyrus (right) (0.177), Precentral Gyrus (left) (0.167), Postcentral Gyrus (left) (0.114) |
|  | IC 40 | Lateral Occipital Cortex, superior division (right) (0.286), Lateral Occipital Cortex, superior division (left) (0.139), Precuneus Cortex (right) (0.106) |
| VI | IC 2 | Lingual Gyrus (right) (0.103), Lingual Gyrus (left) (0.093), Intracalcarine Cortex (right) (0.055) |
|  | IC 4 | Lingual Gyrus (left) (0.122), Lingual Gyrus (right) (0.114), Occipital Fusiform Gyrus (right) (0.112) |
|  | IC 5 | Lateral Occipital Cortex, superior division (right) (0.192), Lateral Occipital Cortex, superior division (left) (0.149), Lateral Occipital Cortex, inferior division (right) (0.049) |
|  | IC 9 | Occipital Pole (right) (0.154), Occipital Pole (left) (0.127), Intracalcarine Cortex (right) (0.082) |
|  | IC 15 | Occipital Pole (left) (0.234), Occipital Pole (right) (0.222), Lateral Occipital Cortex, superior division (right) (0.081) |
|  | IC 17 | Occipital Pole (left) (0.17), Occipital Pole (right) (0.149), Lateral Occipital Cortex, inferior division (right) (0.098) |
|  | IC 42 | Lateral Occipital Cortex, inferior division (right) (0.192), Lateral Occipital Cortex, inferior division (left) (0.135), Middle Temporal Gyrus, temporooccipital part (right) (0.061) |
|  | IC 43 | Lateral Occipital Cortex, inferior division (left) (0.227), Lateral Occipital Cortex, inferior division (right) (0.112), Inferior Temporal Gyrus, temporooccipital part (left) (0.078) |
|  | IC 45 | Temporal Pole (right) (0.109), Temporal Pole (left) (0.087), Inferior Temporal Gyrus, posterior division (right) (0.038) |
|  | IC 60 | Inferior Temporal Gyrus, posterior division (left) (0.156), Crus I Cerebellum (left) (0.123), Inferior Temporal Gyrus, temporooccipital part (left) (0.116) |

**Footnotes:** Bracketed text indicates the overlap degree between the region (e.g., Superior Parietal Lobule (right)) in the atlas and the activation map of each functional network (e.g., IC 35), computed using our NeuroMark toolbox (www.yuhuidu.com).

**Table S2.** Evaluation metrics of the testing data in different outer cross-validation runs for the age prediction using FNC and GMV features. Since in each outer 10-fold cross-validation procedure, the main data set was then further partitioned into 10 folds for the inner cross-validation by employing 9 folds of training data for building the Lasso model and the remaining fold of testing data for evaluating its prediction performance, we include the average, maximum (Max), and minimum (Min) value of evaluation metrics (Corr and MAE) across the 10 runs.

| **Measures** | **Evaluation metrics** | | **Outer CV-1** | **Outer CV-2** | **Outer CV-3** | **Outer CV-4** | **Outer CV-5** | **Outer CV-6** | **Outer CV-7** | **Outer CV-8** | **Outer CV-9** | **Outer CV-10** |
| --- | --- | --- | --- | --- | --- | --- | --- | --- | --- | --- | --- | --- |
| **FNC** | **Corr** | Average | 0.646 | 0.65 | 0.646 | 0.644 | 0.648 | 0.645 | 0.652 | 0.65 | 0.65 | 0.643 |
|  |  | Max | 0.668 | 0.657 | 0.66 | 0.681 | 0.674 | 0.666 | 0.67 | 0.677 | 0.67 | 0.663 |
|  |  | Min | 0.619 | 0.635 | 0.63 | 0.609 | 0.621 | 0.625 | 0.63 | 0.627 | 0.623 | 0.61 |
|  | **MAE** | Average | 5.026 | 5.001 | 5.005 | 5.035 | 4.999 | 5.017 | 4.996 | 4.985 | 4.992 | 5.036 |
|  |  | Max | 5.178 | 5.074 | 5.147 | 5.212 | 5.111 | 5.134 | 5.145 | 5.149 | 5.146 | 5.224 |
|  |  | Min | 4.908 | 4.923 | 4.879 | 4.752 | 4.86 | 4.892 | 4.822 | 4.826 | 4.8 | 4.943 |
| **GMV** | **Corr** | Average | 0.72 | 0.722 | 0.723 | 0.718 | 0.721 | 0.717 | 0.718 | 0.721 | 0.72 | 0.716 |
|  |  | Max | 0.755 | 0.749 | 0.743 | 0.741 | 0.757 | 0.731 | 0.743 | 0.748 | 0.747 | 0.749 |
|  |  | Min | 0.705 | 0.696 | 0.7 | 0.685 | 0.698 | 0.701 | 0.701 | 0.69 | 0.694 | 0.701 |
|  | **MAE** | Average | 4.533 | 4.52 | 4.521 | 4.541 | 4.525 | 4.556 | 4.543 | 4.519 | 4.532 | 4.576 |
|  |  | Max | 4.64 | 4.815 | 4.648 | 4.773 | 4.671 | 4.672 | 4.679 | 4.711 | 4.695 | 4.72 |
|  |  | Min | 4.299 | 4.314 | 4.366 | 4.38 | 4.311 | 4.478 | 4.354 | 4.365 | 4.313 | 4.312 |

**Table S3.** Evaluation metrics of the independent data in different outer cross-validation runs for the age prediction using FNC, GMV, and multimodal features.

| Evaluation metrics | Measures | Outer CV- 1 | Outer CV- 2 | Outer CV- 3 | Outer CV- 4 | Outer CV- 5 | Outer CV- 6 | Outer CV- 7 | Outer CV- 8 | Outer CV- 9 | Outer CV- 10 | Average |
| --- | --- | --- | --- | --- | --- | --- | --- | --- | --- | --- | --- | --- |
| Corr | FNC | 0.645 | 0.673 | 0.654 | 0.665 | 0.640 | 0.634 | 0.653 | 0.655 | 0.635 | 0.639 | 0.649 |
|  | GMV | 0.716 | 0.736 | 0.691 | 0.726 | 0.716 | 0.709 | 0.714 | 0.686 | 0.729 | 0.706 | 0.713 |
|  | Multimodal | 0.780 | 0.800 | 0.772 | 0.781 | 0.778 | 0.764 | 0.772 | 0.771 | 0.779 | 0.762 | 0.776 |
| MAE | FNC | 5.004 | 4.863 | 5.040 | 4.928 | 4.950 | 5.142 | 4.991 | 4.956 | 5.094 | 5.059 | 5.003 |
|  | GMV | 4.577 | 4.430 | 4.789 | 4.517 | 4.614 | 4.577 | 4.586 | 4.729 | 4.467 | 4.670 | 4.596 |
|  | Multimodal | 4.058 | 3.927 | 4.176 | 4.072 | 4.127 | 4.195 | 4.179 | 4.100 | 4.017 | 4.188 | 4.104 |

**Table S4****.** Information of 219 reliable aging-related FNCs. For each FNC, we summarize its two corresponding functional networks, the mean FNC strength across all subjects, the correlation between the mean FNC strength at different ages and chronological ages, and the FNC changing patterns.

| Functional network 1 | Functional network 2 | Mean FNC strength across all subjects | Correlation between the mean FNC strength at different ages and chronological ages | FNC changing pattern |
| --- | --- | --- | --- | --- |
| (Functional domain-IC ID) | (Functional domain-IC ID) |  |  |  |
| VI-IC5 | AU-IC3 | -1.563 | 0.188 | APRN |
| VI-IC5 | VI-IC4 | 2.442 | -0.968 | ANRP |
| DM-IC6 | AU-IC3 | -0.658 | -0.872 | ANRN |
| DM-IC8 | AU-IC3 | -0.241 | 0.882 | APRN |
| DM-IC8 | VI-IC5 | -0.104 | -0.915 | ANRN |
| VI-IC9 | VI-IC4 | 0.469 | -0.803 | ANRP |
| VI-IC9 | DM-IC8 | 0.167 | -0.881 | ANRP |
| AU-IC10 | AU-IC3 | -0.94 | -0.451 | ANRN |
| AU-IC10 | DM-IC8 | -0.134 | -0.019 | ANRN |
| DM-IC11 | AU-IC10 | -1.245 | -0.767 | ANRN |
| CC-IC12 | AU-IC3 | -0.624 | -0.954 | ANRN |
| CC-IC12 | VI-IC9 | -0.196 | -0.801 | ANRN |
| FP-IC13 | VI-IC5 | -0.961 | -0.944 | ANRN |
| FP-IC13 | SM-IC7 | -0.21 | -0.712 | ANRN |
| FP-IC13 | DM-IC8 | -1.305 | 0.69 | APRN |
| FP-IC13 | VI-IC9 | -0.046 | -0.852 | ANRN |
| CC-IC14 | VI-IC5 | -0.279 | -0.645 | ANRN |
| CC-IC14 | DM-IC6 | 2.073 | 0.511 | APRP |
| CC-IC14 | VI-IC9 | 0.256 | -0.898 | ANRP |
| VI-IC15 | AU-IC3 | -0.307 | -0.766 | ANRN |
| VI-IC15 | VI-IC4 | 2.328 | -0.76 | ANRP |
| VI-IC15 | VI-IC5 | 1.297 | 0.977 | APRP |
| VI-IC15 | CC-IC14 | -0.743 | 0.959 | APRN |
| CC-IC16 | SM-IC7 | -0.462 | 0.966 | APRN |
| VI-IC17 | DM-IC6 | -0.032 | -0.428 | ANRN |
| VI-IC17 | AU-IC10 | -0.099 | -0.641 | ANRN |
| CB-IC18 | VI-IC2 | -0.476 | 0.905 | APRN |
| CB-IC18 | DM-IC6 | 0.789 | 0.931 | APRP |
| CB-IC18 | SM-IC7 | -0.351 | -0.923 | ANRN |
| CB-IC18 | AU-IC10 | -0.098 | 0.912 | APRN |
| CB-IC18 | CC-IC14 | 0.738 | 0.564 | APRP |
| CB-IC18 | CC-IC16 | -1.073 | 0.978 | APRN |
| CC-IC19 | CC-IC12 | -1.314 | 0.036 | APRN |
| CC-IC19 | FP-IC13 | -2.267 | 0.885 | APRN |
| CC-IC19 | VI-IC15 | -0.476 | -0.932 | ANRN |
| DM-IC20 | VI-IC4 | -0.133 | -0.901 | ANRN |
| DM-IC20 | DM-IC6 | 0.106 | 0.896 | APRP |
| DM-IC20 | VI-IC9 | -0.272 | 0.906 | APRN |
| DM-IC20 | CC-IC12 | -0.504 | 0.958 | APRN |
| SM-IC21 | DM-IC8 | -0.208 | -0.77 | ANRN |
| SM-IC21 | AU-IC10 | 0.383 | -0.915 | ANRP |
| SM-IC21 | CB-IC18 | -0.456 | -0.893 | ANRN |
| CC-IC22 | VI-IC5 | -0.338 | 0.794 | APRN |
| CC-IC22 | CC-IC14 | 3.423 | -0.391 | ANRP |
| CC-IC22 | VI-IC15 | -0.622 | 0.891 | APRN |
| CC-IC22 | DM-IC20 | -0.828 | 0.938 | APRN |
| AU-IC23 | AU-IC3 | 2.607 | -0.972 | ANRP |
| AU-IC23 | VI-IC4 | -0.004 | 0.879 | APRN |
| AU-IC23 | DM-IC6 | -0.715 | -0.616 | ANRN |
| AU-IC23 | VI-IC9 | -0.248 | -0.589 | ANRN |
| AU-IC23 | CC-IC19 | -0.403 | -0.631 | ANRN |
| AU-IC23 | DM-IC20 | 0.086 | -0.521 | ANRP |
| CB-IC24 | DM-IC6 | -0.049 | -0.798 | ANRN |
| CB-IC24 | VI-IC9 | 0.385 | 0.817 | APRP |
| CB-IC24 | CC-IC14 | -0.132 | -0.962 | ANRN |
| CB-IC24 | CC-IC19 | 0.132 | 0.903 | APRP |
| FP-IC25 | CC-IC16 | -0.49 | 0.298 | APRN |
| CC-IC26 | VI-IC17 | -0.038 | 0.59 | APRN |
| CC-IC26 | CB-IC18 | -0.528 | 0.805 | APRN |
| CC-IC26 | FP-IC25 | 3.651 | 0.289 | APRP |
| CC-IC27 | AU-IC3 | 0.953 | -0.379 | ANRP |
| CC-IC27 | DM-IC8 | -0.692 | -0.909 | ANRN |
| SM-IC28 | AU-IC3 | 2.401 | -0.927 | ANRP |
| SM-IC28 | VI-IC9 | -0.044 | 0.82 | APRN |
| SM-IC28 | SM-IC21 | 1.068 | 0.923 | APRP |
| SM-IC28 | FP-IC25 | -0.852 | 0.614 | APRN |
| CC-IC29 | VI-IC4 | -0.163 | -0.581 | ANRN |
| CC-IC29 | VI-IC5 | -0.12 | -0.789 | ANRN |
| CC-IC29 | FP-IC25 | 0.221 | 0.823 | APRP |
| CC-IC29 | SM-IC28 | 0.771 | -0.905 | ANRP |
| CC-IC30 | CC-IC16 | 1.331 | 0.743 | APRP |
| CC-IC30 | CB-IC18 | 0.058 | 0.946 | APRP |
| CC-IC30 | CC-IC22 | 1.395 | -0.889 | ANRP |
| CC-IC30 | AU-IC23 | -0.13 | 0.778 | APRN |
| SM-IC31 | AU-IC3 | 0.054 | 0.916 | APRP |
| SM-IC31 | VI-IC5 | -0.473 | 0.603 | APRN |
| SM-IC31 | DM-IC20 | -0.393 | 0.958 | APRN |
| SM-IC31 | SM-IC21 | 0.1 | -0.053 | ANRP |
| SM-IC31 | AU-IC23 | 0.125 | 0.943 | APRP |
| CC-IC32 | CB-IC24 | -0.143 | 0.538 | APRN |
| CC-IC32 | FP-IC25 | 2.416 | 0.818 | APRP |
| SM-IC33 | CC-IC22 | -0.163 | 0.933 | APRN |
| SM-IC33 | CB-IC24 | -0.22 | 0.978 | APRN |
| CC-IC34 | AU-IC3 | 0.165 | 0.87 | APRP |
| CC-IC34 | VI-IC15 | -0.155 | -0.683 | ANRN |
| CC-IC34 | DM-IC20 | 0.451 | 0.84 | APRP |
| CC-IC34 | CC-IC22 | 0.814 | -0.909 | ANRP |
| CC-IC34 | AU-IC23 | 0.659 | -0.119 | ANRP |
| AT-IC35 | VI-IC9 | -0.372 | 0.92 | APRN |
| AT-IC35 | CC-IC12 | -0.352 | 0.113 | APRN |
| AT-IC35 | CC-IC19 | 1.92 | 0.9 | APRP |
| AT-IC35 | CC-IC22 | -0.513 | 0.9 | APRN |
| AT-IC35 | CB-IC24 | 0.187 | -0.972 | ANRP |
| AT-IC35 | SM-IC31 | 0.617 | 0.94 | APRP |
| AT-IC35 | SM-IC33 | 0.256 | 0.922 | APRP |
| SM-IC36 | AU-IC3 | 1.698 | -0.797 | ANRP |
| SM-IC36 | DM-IC6 | -0.692 | 0.699 | APRN |
| SM-IC36 | CC-IC12 | -0.648 | -0.692 | ANRN |
| SM-IC36 | VI-IC15 | -0.04 | -0.583 | ANRN |
| SM-IC36 | CB-IC18 | -0.358 | -0.865 | ANRN |
| SM-IC36 | CC-IC19 | -1.539 | 0.992 | APRN |
| SM-IC36 | CC-IC32 | -0.304 | -0.83 | ANRN |
| SM-IC36 | SM-IC33 | 0.35 | -0.909 | ANRP |
| SM-IC36 | AT-IC35 | -0.266 | 0.964 | APRN |
| DM-IC37 | CC-IC14 | -0.939 | 0.813 | APRN |
| DM-IC37 | CC-IC19 | -0.716 | 0.8 | APRN |
| DM-IC37 | CC-IC22 | -0.377 | 0.461 | APRN |
| DM-IC37 | CC-IC26 | -0.657 | 0.906 | APRN |
| DM-IC37 | CC-IC27 | -1.347 | 0.512 | APRN |
| CC-IC38 | SM-IC28 | -0.267 | 0.899 | APRN |
| CC-IC38 | CC-IC32 | 1.36 | -0.509 | ANRP |
| SC-IC39 | AU-IC3 | 0.285 | -0.904 | ANRP |
| SC-IC39 | VI-IC9 | 0.139 | -0.959 | ANRP |
| SC-IC39 | CC-IC14 | -0.242 | -0.251 | ANRN |
| SC-IC39 | CB-IC18 | 0.146 | -0.974 | ANRP |
| SC-IC39 | SM-IC21 | -0.247 | -0.089 | ANRN |
| SC-IC39 | CC-IC34 | 0.066 | -0.839 | ANRP |
| SC-IC39 | AT-IC35 | -0.504 | 0.872 | APRN |
| SM-IC40 | VI-IC5 | 2.801 | -0.918 | ANRP |
| SM-IC40 | SM-IC7 | -0.214 | -0.936 | ANRN |
| SM-IC40 | FP-IC13 | -1.098 | -0.906 | ANRN |
| SM-IC40 | DM-IC20 | 0.93 | -0.802 | ANRP |
| SM-IC40 | SM-IC28 | 0.277 | 0.747 | APRP |
| SM-IC40 | CC-IC30 | 0.306 | -0.053 | ANRP |
| SM-IC40 | SC-IC39 | 0.172 | -0.937 | ANRP |
| CC-IC41 | VI-IC2 | -0.248 | -0.887 | ANRN |
| CC-IC41 | CB-IC18 | -0.577 | 0.763 | APRN |
| CC-IC41 | DM-IC20 | -1.385 | 0.871 | APRN |
| CC-IC41 | SM-IC21 | -0.136 | 0.793 | APRN |
| CC-IC41 | AU-IC23 | -0.463 | -0.892 | ANRN |
| CC-IC41 | CB-IC24 | -0.235 | -0.941 | ANRN |
| CC-IC41 | CC-IC29 | 0.088 | 0.842 | APRP |
| CC-IC41 | DM-IC37 | 0.521 | 0.922 | APRP |
| CC-IC41 | SM-IC40 | 0.131 | 0.885 | APRP |
| VI-IC42 | VI-IC2 | 0.191 | 0.66 | APRP |
| VI-IC42 | VI-IC4 | 0.573 | -0.914 | ANRP |
| VI-IC42 | DM-IC6 | -0.72 | -0.719 | ANRN |
| VI-IC42 | DM-IC8 | 0.155 | -0.87 | ANRP |
| VI-IC42 | CC-IC14 | -0.427 | -0.843 | ANRN |
| VI-IC42 | VI-IC17 | -0.191 | 0.699 | APRN |
| VI-IC42 | AU-IC23 | 0.59 | 0.908 | APRP |
| VI-IC42 | CC-IC30 | -0.437 | -0.2 | ANRN |
| VI-IC42 | SM-IC36 | 0.083 | -0.892 | ANRP |
| VI-IC43 | AU-IC3 | -0.25 | -0.653 | ANRN |
| VI-IC43 | VI-IC4 | 0.445 | -0.66 | ANRP |
| VI-IC43 | VI-IC5 | 1.565 | -0.885 | ANRP |
| VI-IC43 | AU-IC10 | -0.531 | -0.86 | ANRN |
| VI-IC43 | CC-IC12 | 0.028 | -0.961 | ANRP |
| VI-IC43 | VI-IC15 | -0.63 | 0.843 | APRN |
| VI-IC43 | CC-IC19 | 1.937 | -0.915 | ANRP |
| VI-IC43 | DM-IC20 | -0.749 | -0.199 | ANRN |
| VI-IC43 | SM-IC21 | -0.322 | -0.765 | ANRN |
| VI-IC43 | SM-IC28 | -0.891 | 0.951 | APRN |
| VI-IC43 | CC-IC30 | -1.195 | -0.797 | ANRN |
| VI-IC43 | CC-IC34 | -0.583 | -0.027 | ANRN |
| VI-IC45 | CC-IC16 | 0.16 | -0.945 | ANRP |
| VI-IC45 | CB-IC18 | -0.449 | 0.963 | APRN |
| VI-IC45 | CB-IC24 | -0.412 | -0.933 | ANRN |
| VI-IC45 | AT-IC35 | -0.214 | -0.895 | ANRN |
| VI-IC45 | SM-IC36 | -0.047 | -0.919 | ANRN |
| VI-IC45 | SC-IC39 | -0.136 | 0.96 | APRN |
| CC-IC46 | AU-IC3 | 0.28 | 0.88 | APRP |
| CC-IC46 | DM-IC11 | -0.197 | -0.636 | ANRN |
| CC-IC46 | CC-IC22 | 1.429 | -0.842 | ANRP |
| CC-IC46 | CB-IC24 | -0.462 | -0.04 | ANRN |
| CC-IC48 | FP-IC25 | -1.469 | -0.868 | ANRN |
| CC-IC48 | DM-IC37 | 0.657 | 0.877 | APRP |
| CC-IC48 | VI-IC45 | -0.528 | -0.822 | ANRN |
| CC-IC48 | CC-IC46 | -1.518 | 0.958 | APRN |
| AU-IC49 | AU-IC3 | -0.829 | -0.957 | ANRN |
| AU-IC49 | SM-IC7 | -0.379 | -0.455 | ANRN |
| AU-IC49 | VI-IC9 | -0.174 | -0.791 | ANRN |
| AU-IC49 | CB-IC18 | 0.782 | -0.333 | ANRP |
| AU-IC49 | CC-IC19 | -0.768 | 0.753 | APRN |
| AU-IC49 | SM-IC21 | -0.063 | -0.745 | ANRN |
| AU-IC49 | SM-IC36 | 0.01 | -0.961 | ANRP |
| AU-IC49 | VI-IC45 | -0.085 | 0.935 | APRN |
| AU-IC49 | CC-IC48 | -0.17 | 0.288 | APRN |
| CC-IC50 | AU-IC3 | 0.183 | 0.799 | APRP |
| CC-IC50 | CC-IC16 | 1.429 | 0.587 | APRP |
| CC-IC50 | CB-IC18 | 0.087 | -0.974 | ANRP |
| CC-IC50 | SM-IC28 | 0.086 | 0.875 | APRP |
| CC-IC50 | SM-IC31 | -0.248 | 0.87 | APRN |
| CC-IC50 | SM-IC33 | -0.158 | 0.927 | APRN |
| CC-IC50 | CC-IC41 | 0.478 | 0.858 | APRP |
| CC-IC50 | CC-IC46 | -0.908 | 0.969 | APRN |
| CC-IC50 | AU-IC49 | -0.354 | 0.283 | APRN |
| AT-IC52 | AU-IC23 | 0.872 | 0.63 | APRP |
| DM-IC53 | VI-IC4 | -0.552 | -0.761 | ANRN |
| DM-IC53 | DM-IC11 | 2.313 | 0.385 | APRP |
| DM-IC53 | CC-IC34 | 1.149 | -0.863 | ANRP |
| DM-IC53 | AT-IC35 | 0.217 | 0.861 | APRP |
| DM-IC53 | AT-IC52 | 1.839 | -0.209 | ANRP |
| CC-IC57 | SM-IC7 | 0.002 | 0.859 | APRP |
| CC-IC57 | VI-IC9 | -0.338 | 0.945 | APRN |
| CC-IC57 | AU-IC23 | 0.045 | 0.787 | APRP |
| CC-IC57 | AU-IC49 | 0.457 | 0.927 | APRP |
| CB-IC58 | VI-IC5 | -0.625 | -0.26 | ANRN |
| CB-IC58 | VI-IC9 | 0.465 | -0.93 | ANRP |
| CB-IC58 | VI-IC17 | 0.325 | -0.941 | ANRP |
| CB-IC58 | CB-IC18 | -0.345 | 0.948 | APRN |
| CB-IC58 | CB-IC24 | 0.129 | 0.909 | APRP |
| CB-IC58 | CC-IC27 | -0.147 | 0.86 | APRN |
| CB-IC58 | CC-IC41 | -0.342 | -0.727 | ANRN |
| VI-IC60 | VI-IC5 | -0.113 | -0.631 | ANRN |
| VI-IC60 | DM-IC6 | -0.115 | -0.693 | ANRN |
| VI-IC60 | AU-IC10 | -0.084 | -0.923 | ANRN |
| VI-IC60 | FP-IC13 | -0.304 | 0.874 | APRN |
| VI-IC60 | VI-IC15 | 0.419 | -0.89 | ANRP |
| VI-IC60 | CC-IC19 | 0.068 | 0.895 | APRP |
| VI-IC60 | DM-IC20 | -0.249 | -0.571 | ANRN |
| CC-IC63 | VI-IC9 | 0.101 | -0.79 | ANRP |
| CC-IC63 | CC-IC14 | -0.173 | 0.938 | APRN |
| CC-IC63 | CB-IC18 | -0.116 | 0.283 | APRN |
| CC-IC64 | CC-IC12 | -0.23 | -0.608 | ANRN |
| CC-IC64 | VI-IC15 | -0.1 | -0.765 | ANRN |
| CC-IC64 | VI-IC45 | -0.151 | 0.77 | APRN |
| CC-IC64 | VI-IC60 | -0.56 | 0.955 | APRN |
| CC-IC93 | SM-IC40 | -0.583 | 0.835 | APRN |

**Table S5** Information of 96 reliable aging-related GMVs. For each GMV, we include its corresponding region name in the atlas, the mean GMV value across all subjects, the correlation between the mean GMV value at different ages and chronological ages, and the GMV changing patterns.

| Brain region name | Mean GMV value across all subjects | Correlation between the mean GMV value at different ages and chronological ages | GMV changing pattern |
| --- | --- | --- | --- |
| Frontal Pole (left) | 23285.156 | -0.989 | AN |
| Frontal Pole (right) | 26369.676 | -0.983 | AN |
| Insular Cortex (left) | 6330.420 | -0.973 | AN |
| Insular Cortex (right) | 6313.516 | -0.965 | AN |
| Superior Frontal Gyrus (left) | 11103.693 | -0.976 | AN |
| Superior Frontal Gyrus (right) | 9660.402 | -0.973 | AN |
| Middle Frontal Gyrus (left) | 10094.425 | -0.964 | AN |
| Middle Frontal Gyrus (right) | 9585.555 | -0.968 | AN |
| Inferior Frontal Gyrus, pars triangularis (left) | 2451.620 | -0.986 | AN |
| Inferior Frontal Gyrus, pars opercularis (left) | 2658.592 | -0.986 | AN |
| Inferior Frontal Gyrus, pars opercularis (right) | 2481.750 | -0.981 | AN |
| Precentral Gyrus (left) | 13915.917 | -0.995 | AN |
| Temporal Pole (left) | 9488.168 | -0.992 | AN |
| Superior Temporal Gyrus, anterior division (left) | 1474.736 | -0.983 | AN |
| Superior Temporal Gyrus, anterior division (right) | 1478.992 | -0.988 | AN |
| Middle Temporal Gyrus, anterior division (right) | 1618.326 | -0.967 | AN |
| Middle Temporal Gyrus, posterior division (left) | 5346.034 | -0.981 | AN |
| Middle Temporal Gyrus, posterior division (right) | 5514.000 | -0.993 | AN |
| Middle Temporal Gyrus, temporooccipital part (left) | 3495.174 | -0.931 | AN |
| Middle Temporal Gyrus, temporooccipital part (right) | 4725.458 | -0.955 | AN |
| Inferior Temporal Gyrus, anterior division (left) | 1440.936 | -0.969 | AN |
| Inferior Temporal Gyrus, posterior division (left) | 4139.569 | -0.962 | AN |
| Inferior Temporal Gyrus, temporooccipital part (left) | 3014.414 | -0.73 | AN |
| Inferior Temporal Gyrus, temporooccipital part (right) | 3836.766 | -0.846 | AN |
| Postcentral Gyrus (left) | 11234.363 | -0.995 | AN |
| Postcentral Gyrus (right) | 10495.995 | -0.995 | AN |
| Superior Parietal Lobule (left) | 5147.332 | -0.982 | AN |
| Supramarginal Gyrus, anterior division (left) | 3206.289 | -0.959 | AN |
| Supramarginal Gyrus, anterior division (right) | 3147.527 | -0.95 | AN |
| Supramarginal Gyrus, posterior division (right) | 5490.594 | -0.982 | AN |
| Angular Gyrus (left) | 4083.187 | -0.869 | AN |
| Lateral Occipital Cortex, superior division (right) | 15757.448 | -0.992 | AN |
| Lateral Occipital Cortex, inferior division (left) | 7232.628 | -0.96 | AN |
| Intracalcarine Cortex (left) | 2612.240 | -0.982 | AN |
| Intracalcarine Cortex (right) | 2730.177 | -0.977 | AN |
| Frontal Medial Cortex (left) | 1888.271 | -0.983 | AN |
| Juxtapositional Lobule Cortex (formerly Supplementary Motor Cortex) (right) | 2784.946 | -0.992 | AN |
| Subcallosal Cortex (left) | 2951.348 | -0.984 | AN |
| Subcallosal Cortex (right) | 2731.296 | -0.897 | AN |
| Paracingulate Gyrus (right) | 5709.322 | -0.995 | AN |
| Cingulate Gyrus, anterior division (right) | 5644.281 | 0.566 | AP |
| Cingulate Gyrus, posterior division (right) | 5441.134 | -0.863 | AN |
| Precuneus Cortex (right) | 10569.223 | -0.992 | AN |
| Cuneal Cortex (right) | 2326.506 | -0.981 | AN |
| Frontal Orbital Cortex (left) | 6641.571 | -0.994 | AN |
| Frontal Orbital Cortex (right) | 6042.414 | -0.989 | AN |
| Parahippocampal Gyrus, anterior division (right) | 3020.098 | -0.978 | AN |
| Lingual Gyrus (left) | 6557.476 | -0.974 | AN |
| Temporal Fusiform Cortex, anterior division (right) | 1382.318 | -0.993 | AN |
| Temporal Fusiform Cortex, posterior division (right) | 3258.887 | -0.987 | AN |
| Temporal Occipital Fusiform Cortex (left) | 2636.280 | -0.975 | AN |
| Temporal Occipital Fusiform Cortex (right) | 3359.080 | -0.971 | AN |
| Occipital Fusiform Gyrus (left) | 3932.776 | -0.991 | AN |
| Frontal Operculum Cortex (left) | 1516.218 | -0.991 | AN |
| Frontal Operculum Cortex (right) | 1345.505 | -0.985 | AN |
| Central Opercular Cortex (left) | 3790.761 | -0.99 | AN |
| Planum Polare (left) | 1396.752 | -0.985 | AN |
| Planum Polare (right) | 1476.010 | -0.992 | AN |
| Heschl's Gyrus (includes H1 and H2) (left) | 1197.821 | -0.992 | AN |
| Heschl's Gyrus (includes H1 and H2) (right) | 1036.746 | -0.994 | AN |
| Planum Temporale (right) | 1625.437 | -0.976 | AN |
| Occipital Pole (left) | 8575.939 | -0.983 | AN |
| Thalamus (left) | 2703.721 | -0.784 | AN |
| Thalamus (right) | 2832.275 | 0.831 | AP |
| Caudate (left) | 3015.187 | 0.894 | AP |
| Caudate (right) | 3230.880 | 0.905 | AP |
| Putamen (left) | 1763.204 | 0.487 | AP |
| Putamen (right) | 2185.391 | -0.492 | AN |
| Pallidum (left) | 40.191 | 0.983 | AP |
| Amygdala (left) | 1844.554 | -0.979 | AN |
| Ventral Striatum (left) | 561.108 | -0.986 | AN |
| Brain-Stem | 4913.549 | -0.989 | AN |
| VI Cerebellum (left) | 6846.121 | -0.972 | AN |
| VI Cerebellum (vermis) | 1521.585 | -0.926 | AN |
| Crus I Cerebellum (vermis) | 2.023 | -0.916 | AN |
| Crus I Cerebellum (right) | 11572.837 | -0.994 | AN |
| Crus II Cerebellum (vermis) | 405.743 | -0.785 | AN |
| Crus II Cerebellum (right) | 7891.812 | -0.987 | AN |
| VIIb Cerebellum (left) | 3927.462 | -0.942 | AN |
| VIIIa Cerebellum (left) | 3875.618 | -0.921 | AN |
| VIIIa Cerebellum (vermis) | 915.378 | -0.926 | AN |
| VIIIb Cerebellum (left) | 2719.237 | -0.874 | AN |
| VIIIb Cerebellum (vermis) | 449.506 | -0.89 | AN |
| VIIIb Cerebellum (right) | 2767.570 | -0.899 | AN |
| IX Cerebellum (left) | 1922.698 | -0.762 | AN |
| IX Cerebellum (right) | 2164.012 | -0.8 | AN |
| X Cerebellum (vermis) | 233.848 | -0.86 | AN |
| X Cerebellum (right) | 456.533 | -0.993 | AN |
| Subcortical caudate (left) | 3385.923 | -0.922 | AN |
| Subcortical putamen (left) | 4790.718 | -0.994 | AN |
| Subcortical pallidum (left) | 1761.967 | -0.931 | AN |
| Subcortical pallidum (right) | 1809.712 | -0.948 | AN |
| Subcortical hippocampus (left) | 3804.532 | -0.975 | AN |
| Subcortical amygdala (left) | 1264.491 | -0.383 | AN |
| Subcortical amygdala (right) | 1228.881 | -0.413 | AN |
| Subcortical accumbens (right) | 394.300 | -0.984 | AN |

**Table S6.** Information of the significant joint changes. For each joint change, we display its relevant FNC and two GMVs, changing pattern, and the correlation between each of the three neuroimage measures (i.e., FNC and two GMVs) and each of the three cognitive scores (i.e., FI, NM, and RT).

| **Relevant FNC and GMVs of each significant joint change** | **Joint, FNC, and GMV changing pattern** | **FI** | **NM** | **RT** |
| --- | --- | --- | --- | --- |
|  |  | **r1, r2, r3, Mean of abs (r)** | **r1, r2, r3, Mean of abs (r)** | **r1, r2, r3, Mean of abs (r)** |
| FNC: CB-IC18 and CC-IC16 GMV1: GMV of Crus I Cerebellum (right) GMV2: GMV of Paracingulate Gyrus (right) | Synergistic change, APRN, AN | -0.586; 0.615; 0.644; 0.615 | -0.798; 0.846; 0.861; 0.835 | **0.941; -0.972; -0.980; 0.964** |
| FNC: CC-IC16 and SM-IC7 GMV1: GMV of Paracingulate Gyrus (right) GMV2: GMV of Precentral Gyrus (left) | Synergistic change, APRN, AN | -0.5; 0.644; 0.562; 0.569 | -0.774; 0.861; 0.813; 0.816 | 0.942; -0.98; -0.966; 0.963 |
| FNC: VI-IC15 and VI-IC5 GMV1: GMV of Occipital Pole (left) GMV2: GMV of Lateral Occipital Cortex, superior division (right) | Contradictory change, APRP, AN | **-0.684; 0.655; 0.694; 0.678** | **-0.853; 0.867; 0.867; 0.862** | 0.938; -0.973; -0.955; 0.956 |
| FNC: CC-IC50 and CB-IC18 GMV1: GMV of Frontal Pole (right) GMV2: GMV of Crus I Cerebellum (right) | Synergistic change, ANRP, AN | 0.626; 0.554; 0.615; 0.599 | 0.853; 0.812; 0.846; 0.837 | -0.932; -0.962; -0.972; 0.955 |
| FNC: SM-IC33 and CB-IC24 GMV1: GMV of Postcentral Gyrus (right) GMV2: GMV of VI Cerebellum (left) | Synergistic change, APRN, AN | -0.518; 0.622; 0.719; 0.619 | -0.783; 0.851; 0.914; 0.849 | 0.945; -0.98; -0.959; 0.962 |
| FNC: CC-IC50 and CC-IC46 GMV1: GMV of Frontal Pole (right) GMV2: GMV of Frontal Pole (right) | Synergistic change, APRN, AN | -0.461; 0.554; 0.554; 0.523 | -0.71; 0.812; 0.812; 0.778 | 0.901; -0.962; -0.962; 0.942 |
| FNC: DM-IC20 and CC-IC12 GMV1: GMV of Precuneus Cortex (right) GMV2: GMV of Frontal Pole (right) | Synergistic change, APRN, AN | -0.561; 0.603; 0.554; 0.572 | -0.819; 0.831; 0.812; 0.821 | 0.943; -0.966; -0.962; 0.957 |
| FNC: VI-IC15 and CC-IC14 GMV1: GMV of Occipital Pole (left) GMV2: GMV of Frontal Pole (left) | Synergistic change, APRN, AN | -0.526; 0.655; 0.56; 0.581 | -0.753; 0.867; 0.825; 0.815 | 0.858; -0.973; -0.963; 0.931 |
| FNC: CC-IC48 and CC-IC46 GMV1: GMV of Frontal Pole (right) GMV2: GMV of Frontal Pole (right) | Synergistic change, APRN, AN | -0.582; 0.554; 0.554; 0.563 | -0.796; 0.812; 0.812; 0.807 | 0.874; -0.962; -0.962; 0.933 |
| FNC: CB-IC24 and CC-IC14 GMV1: GMV of VI Cerebellum (left) GMV2: GMV of Frontal Pole (left) | Contradictory change, ANRN, AN | 0.394; 0.719; 0.56; 0.558 | 0.672; 0.914; 0.825; 0.803 | -0.884; -0.959; -0.963; 0.935 |
| FNC: SM-IC40 and SM-IC7 GMV1: GMV of Lateral Occipital Cortex, superior division (right) GMV2: GMV of Precentral Gyrus (left) | Contradictory change, ANRN, AN | 0.626; 0.694; 0.562; 0.628 | 0.758; 0.867; 0.813; 0.813 | -0.829; -0.955; -0.966; 0.917 |
| FNC: FP-IC13 and VI-IC5 GMV1: GMV of Frontal Pole (right) GMV2: GMV of Lateral Occipital Cortex, superior division (right) | Contradictory change, ANRN, AN | 0.454; 0.554; 0.694; 0.567 | 0.744; 0.812; 0.867; 0.807 | -0.847; -0.962; -0.955; 0.921 |
| FNC: CB-IC18 and SM-IC7 GMV1: GMV of Crus I Cerebellum (right) GMV2: GMV of Precentral Gyrus (left) | Contradictory change, ANRN, AN | 0.407; 0.615; 0.562; 0.528 | 0.654; 0.846; 0.813; 0.771 | -0.863; -0.972; -0.966; 0.934 |
| FNC: SM-IC33 and CC-IC22 GMV1: GMV of Postcentral Gyrus (right) GMV2: GMV of Frontal Pole (right) | Synergistic change, APRN, AN | -0.453; 0.622; 0.554; 0.543 | -0.7; 0.851; 0.812; 0.788 | 0.778; -0.98; -0.962; 0.907 |
| FNC: CC-IC19 and VI-IC15 GMV1: GMV of Precentral Gyrus (left) GMV2: GMV of Occipital Pole (left) | Contradictory change, ANRN, AN | 0.487; 0.562; 0.655; 0.568 | 0.686; 0.813; 0.867; 0.789 | -0.801; -0.966; -0.973; 0.913 |

**Footnotes:** "r1" represents the correlation between the mean values of the FNC at different ages and the mean values of each cognitive score at different ages. "r2" represents the correlation between the mean values of the GMV1 at different ages and the mean values of each cognitive score at different ages. "r3" represents the correlation between the mean values of the GMV2 at different ages and the mean values of each cognitive score at different ages. The two GMVs are associated with the FNC after matching them according to the same atlas. "Mean of abs(r)" represents the mean of the absolute values of the three correlations (r1, r2, r3). **Bold text** indicates the maximum mean value of absolute correlations across all significant joint changes for each cognitive score.

**Table S7.** Number of participants by primary education level and age group.

| **Age** | **Total number** | **Number of O levels**  **(percentage)** | **Number of A levels (percentage)** | **Number of College or University degree (percentage)** |
| --- | --- | --- | --- | --- |
| 49 | 352 | 71 (20.17%) | 65 (18.47%) | 170 (48.30%) |
| 50 | 501 | 104 (20.76%) | 78 (15.57%) | 242 (48.30%) |
| 51 | 691 | 131 (18.96%) | 93 (13.46%) | 368 (53.26%) |
| 52 | 841 | 150 (17.84%) | 119 (14.15%) | 450 (53.51%) |
| 53 | 916 | 161 (17.58%) | 130 (14.19%) | 491 (53.60%) |
| 54 | 926 | 180 (19.44%) | 138 (14.90%) | 463 (50.00%) |
| 55 | 1034 | 218 (21.08%) | 143 (13.83%) | 494 (47.78%) |
| 56 | 974 | 179 (18.38%) | 151 (15.50%) | 475 (48.77%) |
| 57 | 999 | 197 (19.72%) | 165 (16.52%) | 492 (49.25%) |
| 58 | 1040 | 195 (18.75%) | 156 (15.00%) | 518 (49.81%) |
| 59 | 1122 | 215 (19.16%) | 178 (15.86%) | 562 (50.09%) |
| 60 | 1133 | 228 (20.12%) | 171 (15.09%) | 548 (48.37%) |
| 61 | 1231 | 191 (15.52%) | 198 (16.08%) | 602 (48.90%) |
| 62 | 1247 | 202 (16.20%) | 177 (14.19%) | 664 (53.25%) |
| 63 | 1240 | 224 (18.06%) | 185 (14.92%) | 603 (48.63%) |
| 64 | 1230 | 219 (17.80%) | 155 (12.60%) | 597 (48.54%) |
| 65 | 1294 | 199 (15.38%) | 187 (14.45%) | 665 (51.39%) |
| 66 | 1315 | 254 (19.32%) | 147 (11.18%) | 624 (47.45%) |
| 67 | 1330 | 227 (17.07%) | 143 (10.75%) | 664 (49.92%) |
| 68 | 1267 | 205 (16.18%) | 155 (12.23%) | 609 (48.07%) |
| 69 | 1292 | 245 (18.96%) | 136 (10.53%) | 584 (45.20%) |
| 70 | 1261 | 267 (21.17%) | 131 (10.39%) | 565 (44.81%) |
| 71 | 1110 | 203 (18.29%) | 119 (10.72%) | 494 (44.50%) |
| 72 | 982 | 195 (19.86%) | 107 (10.90%) | 423 (43.08%) |
| 73 | 831 | 153 (18.41%) | 83 (9.99%) | 367 (44.16%) |
| 74 | 639 | 119 (18.62%) | 68 (10.64%) | 275 (43.04%) |
| 75 | 568 | 110 (19.37%) | 58 (10.21%) | 231 (40.67%) |
| 76 | 427 | 78 (18.27%) | 41 (9.60%) | 175 (40.98%) |


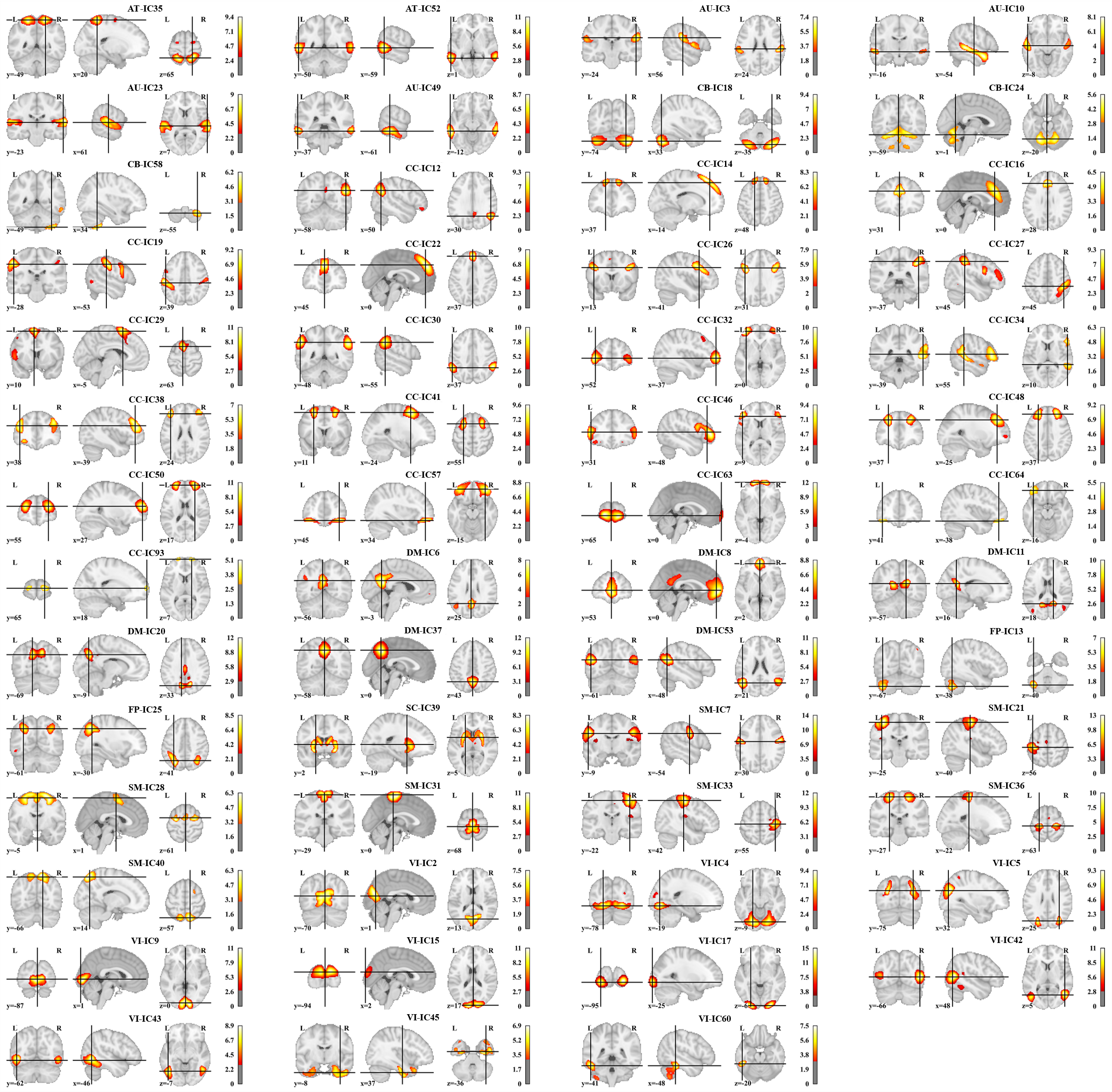


**Fig. S1| Spatial maps of 55 functional networks that were estimated from the UK Biobank datasets using a group ICA.** All these functional networks were assigned into nine functional domains including attentional (AT), auditory (AU), cerebellum (CB), cognitive control (CC), default mode (DM), frontoparietal (FP), subcortical (SC), sensorimotor (SM), and visual (VI) domains. For each network, the IC ID and its corresponding functional domain are shown above the network map.


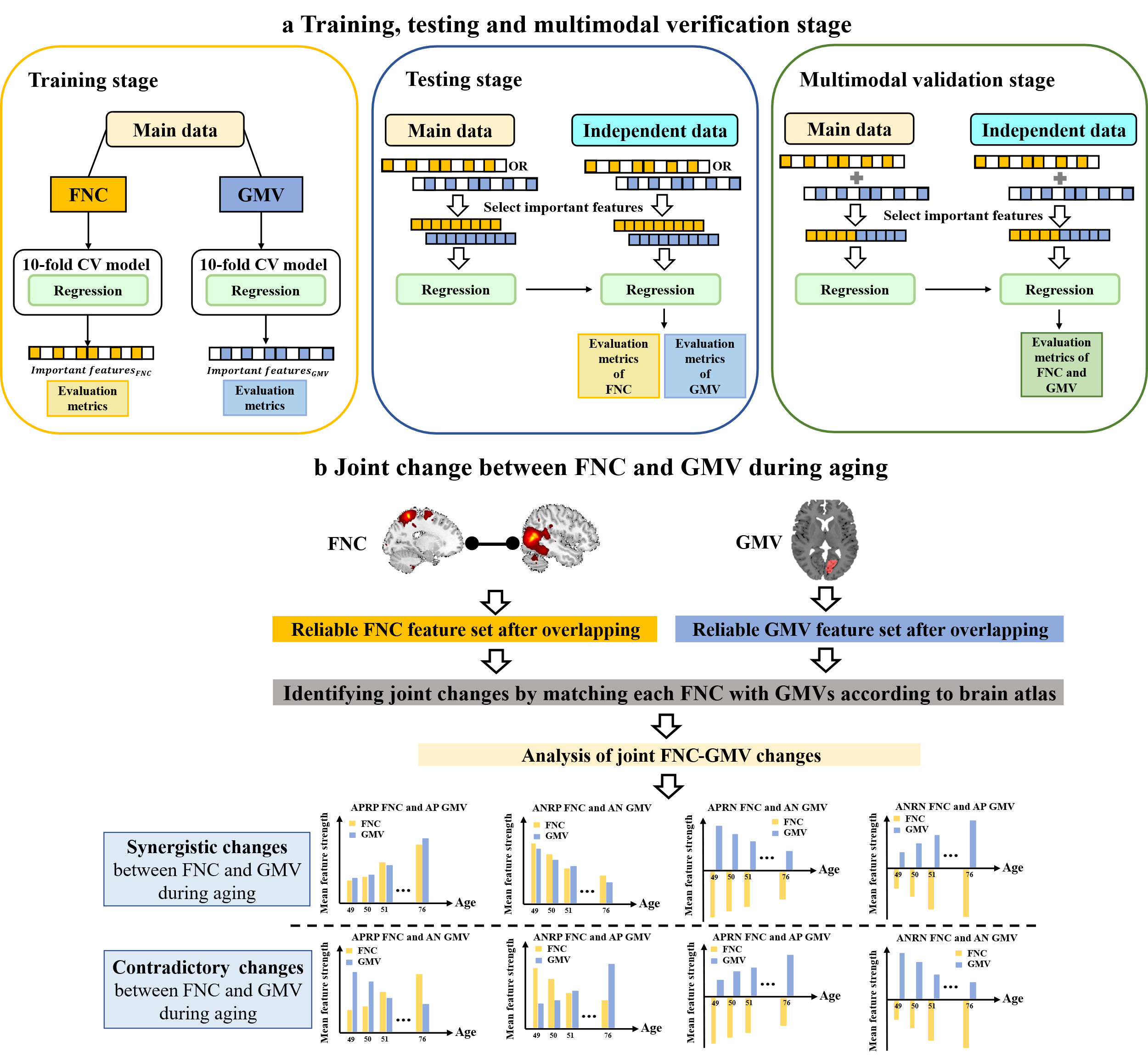


**Fig. S2| Multimodal joint analysis framework related to aging.** **a,** Training, testing and multimodal verification stage. **b,** Joint change between FNC and GMV during aging.
